# Arachidonate lipoxygenase 5 metabolism axis promoting ferroptosis: a potential druggable target for doxorubicin-induced cardiomyopathy

**DOI:** 10.1038/s41416-026-03376-3

**Published:** 2026-04-06

**Authors:** Lu Chen, Xingang Sun, Han Zhang, Xuan Zhang, Mairedan Muhetarijiang, Zuoshi Wen, Chenxi Li, Mengjia Chen, Zhangquan Ying, Shuai Yuan, Liangrong Zheng, Ting Chen

**Affiliations:** 1 Department of Cardiology, The First Affiliated Hospital, School of Medicine, Zhejiang University, Hangzhou, Zhejiang Province China; 2 Department of Critical Care Medicine, Sir Run Run Shaw Hospital, School of Medicine, Zhejiang University, Hangzhou, Zhejiang Province China; 3 Department of Cardiology, Zhejiang Provincial People’s Hospital, People’s Hospital of Hangzhou Medical College, Hangzhou, Zhejiang Province China; 4 Department of Echocardiography and Vascular Ultrasound Center, The First Affiliated Hospital, School of Medicine, Zhejiang University, Hangzhou, Zhejiang Province China

**Keywords:** Cardiomyopathies, Heart failure

## Abstract

**Background:**

Doxorubicin (Dox) is a widely-used anthracycline drug for various cancers, but its clinical application is limited due to cardiotoxicity. Targeting ferroptosis is a new and effective strategy for treating Dox-induced cardiomyopathy (DIC). The arachidonate lipoxygenase 5 (ALOX5) metabolism axis is closely linked to ferroptosis, but its role in DIC remains unknown.

**Methods:**

In vivo and in vitro DIC models were established to investigate the role of the ALOX5 metabolism axis in DIC and to clarify the underlying mechanism using genetic and pharmacological approaches.

**Results:**

Our findings revealed that ALOX5 and 5-hydroxyicosatetraenoic acid (5-HETE) levels were increased in DIC. Overexpression of *Alox5* exacerbated Dox-induced cardiac dysfunction and myocardial injury, whereas pharmacological inhibition and genetic knockdown of ALOX5 alleviated these effects by modulating ferroptosis. Mechanistically, elevated ALOX5 catalyzed the metabolism of arachidonic acid to generate 5-HETE, which facilitated NRF2 ubiquitination-dependent degradation via PI3K/AKT/GSK-3β signaling, thereby contributing to cardiomyocyte ferroptosis.

**Conclusions:**

This study suggests that targeting cardiomyocyte ferroptosis mediated by the ALOX5 metabolism axis may represent a therapeutic strategy for DIC.

## Introduction

Doxorubicin (Dox) is one of the most widely used anthracycline drugs effective in treating a broad spectrum of hematological malignancies and solid tumors. However, the clinical application of Dox is limited by its cumulative and dose-dependent cardiotoxic side effects [1]. To date, effective and safe strategies for the prevention and treatment of Dox-induced cardiomyopathy (DIC) remain scarce.

The precise mechanism of DIC remains elusive, with the involvement of various forms of cardiomyocyte death, including apoptosis, autophagy, necroptosis, and ferroptosis [2]. Among these, ferroptosis is a novel form of regulated cell death [3], characterized by iron overload, intracellular lipid peroxidation, and disruption of the glutathione peroxidase 4 (GPX4)/glutathione (GSH) antioxidant system [4–6]. Recently, a growing number of studies have shown that ferroptosis plays a pivotal role in the development of DIC [7, 8]. Notably, in contrast to interventions targeting other forms of cell death, inhibition of ferroptosis significantly alleviated Dox-induced mortality in mice, suggesting a predominant role of ferroptosis in DIC [8]. Therefore, developing targeted medications against ferroptosis represents a promising therapeutic strategy to prevent and attenuate DIC.

Arachidonate lipoxygenase 5 (ALOX5), a member of the lipoxygenases (LOXs) family, primarily metabolizes arachidonic acid (AA) to 5-hydroperoxyeicostetraenoic acid, which is further converted to 5-hydroxyicosatetraenoic acid (5-HETE) and leukotriene [9]. Despite the established roles of ALOX5 in several cardiovascular diseases and its emerging link to ferroptosis, the function of the ALOX5 metabolism axis in DIC, particularly its specific regulatory mechanisms in ferroptosis, remains elusive [10–15]. Therefore, this study established both in vivo and in vitro DIC models to investigate the role of the ALOX5 metabolism axis in DIC and to elucidate its underlying mechanism using genetic and pharmacological approaches.

## Methods

### Please see the Supplementary Materials for a more detailed description of the methods employed in this study

Statistical analysis was performed utilizing GraphPad Prism 10.2.0 software (GraphPad Software, San Diego, CA, USA). Data were presented as mean ± SD. Data normality was assessed using the Shapiro-Wilk test. For comparisons between two groups, the F-test was used to evaluate variance homogeneity, and the Student’s t-test was applied when variances were similar; otherwise, Welch’s correction was applied. For comparisons among multiple groups, the Brown-Forsythe test was applied to assess the homogeneity of variances. The ordinary one-way ANOVA analysis was conducted for the data with similar variances; otherwise, the Welch ANOVA analysis was applied. In this study, *P* < 0.05 was deemed statistically significant.

## Results

### ALOX5 expression is upregulated in DIC

**Fig. 1:**
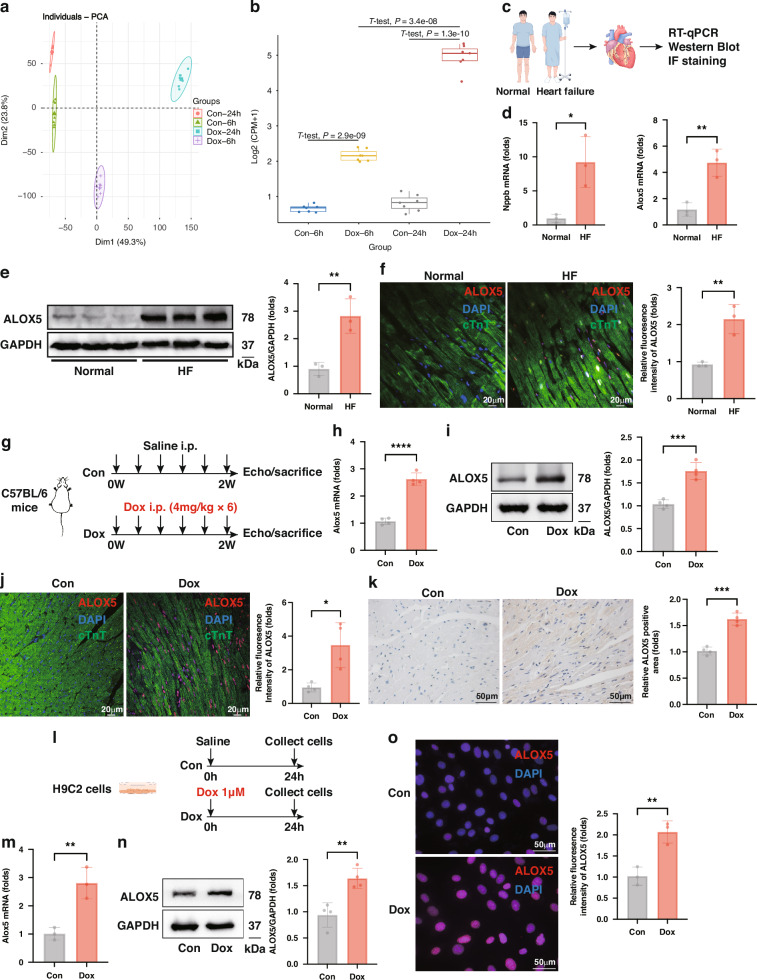
ALOX5 is upregulated in human HF hearts and in vivo and in vitro models of DIC. **a** The principal component analysis showed that the gene expression patterns were consistent within each group but distinct between groups. **b** Expression of *Alox5* in NRCMs treated with Dox or DMSO for 6 h or 24 h in the GSE166957 dataset. **c** Experimental design for assessing the ALOX5 expression in human HF hearts. **d** The mRNA level of *Nppb* and *Alox5* (*n* = 3). **e**, **f** Representative WB and IF images, and quantitative analysis of ALOX5 protein level (*n* = 3). **g** Experimental design for exploring the ALOX5 expression in the in vivo DIC model. **h** The *Alox5* mRNA level in murine heart tissues (*n* = 4). **i**–**k** Representative WB, IF, and IHC images, and the quantitative analysis of ALOX5 protein level in murine heart tissues (*n* = 4). **l** Experimental design for exploring the expression of ALOX5 in the in vitro DIC model. **m** The *Alox5* mRNA level in H9C2 cells (*n* = 3). **n** WB analysis of ALOX5 protein level in H9C2 cells (*n* = 4). **o** Representative IF staining images and quantitative analysis of ALOX5 protein level in H9C2 cells (*n* = 3). *P < 0.05; **P < 0.01; ***P < 0.001; ****P < 0.0001. HF heart failure, DIC doxorubicin-induced cardiomyopathy, NRCMs neonatal rat cardiomyocytes, Dox doxorubicin, WB Western Blot, IF immunofluorescence, IHC immunohistochemistry.

Initially, the sequencing data using mRNA extracted from neonatal rat cardiomyocytes (NRCMs) treated with Dox or Dimethyl Sulfoxide (DMSO) for either 6 or 24 h were analysed to explore the expression of ALOX5 in DIC. This dataset is accessible in the Gene Expression Omnibus (GEO) database under accession number GSE166957. Correlation analysis (Figure S1A), cluster analysis (Figure S1B), and principal component analysis (Fig. 1a) were performed, confirming that the gene expression patterns were consistent within each group but distinct between groups. A total of 509 differentially expressed genes (DEGs) were identified between NRCMs treated with Dox for 6 h and those treated with DMSO for the same duration. When the treatment was extended to 24 h, 2196 DEGs were found between the Dox- and DMSO-treated NRCMs. These DEGs were then intersected with ferroptosis-related genes (FRGs), as illustrated in the Venn diagram (Figure S1C), revealing a total of 8 differentially expressed ferroptosis-related genes (DEFRGs). The volcano plot displays the changes in these 8 genes (Figure S1D). Notably, *Alox5* was upregulated in DIC and ranked among the top 3 DEFRGs. As depicted in Fig. 1b, the expression of *Alox5* was upregulated in NRCMs treated with Dox for either 6 or 24 h, with a more pronounced increase in the 24-h group compared to the 6-h group.

Furthermore, the expression of ALOX5 in the cardiac tissues of heart failure (HF) patients and normal controls was evaluated (Fig. 1c). Real-time quantitative PCR (RT-qPCR) analysis indicated that the mRNA levels of the HF biomarker *Nppb* and *Alox5* were higher in HF heart tissues compared with normal controls (Fig. 1d). Western Blot (WB) and immunofluorescence (IF) assays showed that the protein level of ALOX5 was significantly increased in HF patients (Fig. 1e, f). Subsequently, in vivo (Fig. 1g) and in vitro (Fig. 1l) DIC models were established to assess ALOX5 expression in the DIC heart tissues and cardiomyocytes. The mRNA and protein levels of ALOX5 were significantly upregulated in the heart tissues of Dox-treated mice compared with the control mice (Fig. 1h–k). Moreover, Dox administration resulted in significant increases in the mRNA and protein levels of ALOX5 in H9C2 cells (Fig. 1m–o). Overall, these results indicate that ALOX5 expression is increased in DIC, and ALOX5 may participate in the pathogenesis of DIC.

### Cardiomyocyte-specific overexpression of *Alox5* aggravates DIC by ‌promoting ferroptosis in vivo

**Fig. 2:**
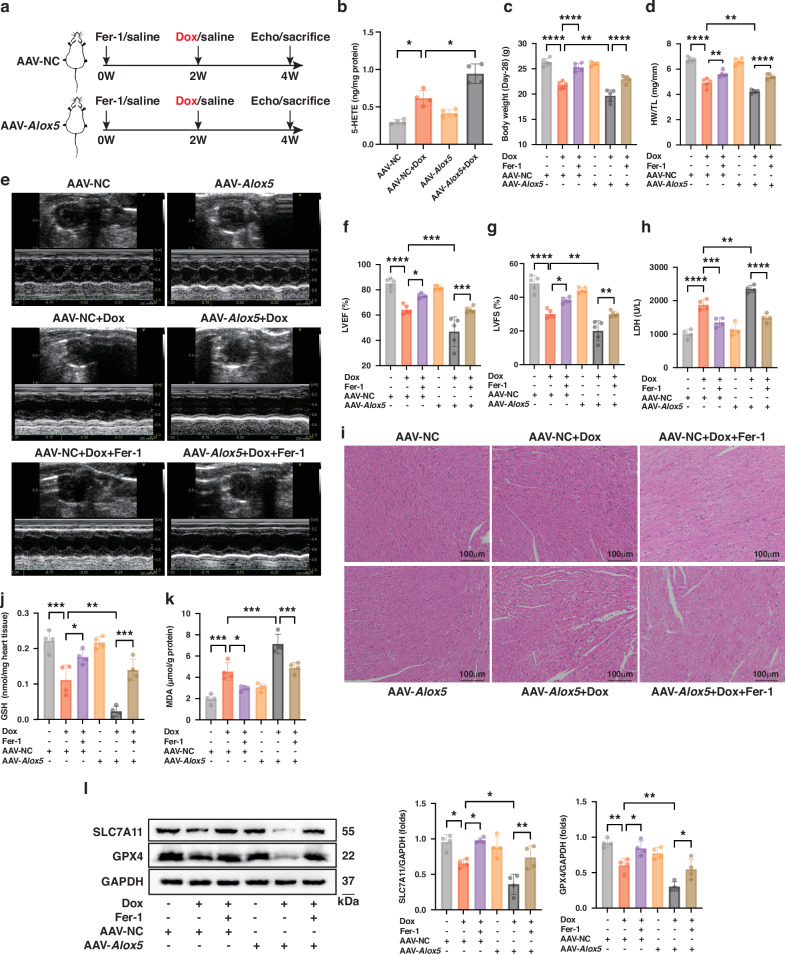
Cardiomyocyte-specific overexpression of *Alox5* aggravates DIC by ‌promoting ferroptosis in vivo. **a** Experimental design for investigating the effect of cardiomyocyte-specific *Alox5* overexpression on DIC in vivo. **b** 5-HETE content in murine heart tissues (*n* = 4). **c**, **d** Body weight and HW/TL of mice (*n* = 5). **e**–**g** Representative cardiac echocardiography images and quantitative analysis of LVEF and LVFS in mice (*n* = 5). **h** Serum LDH levels (*n* = 4). **i** Representative HE staining images of the heart tissues (*n* = 3). **j**, **k** GSH content and MDA levels in the heart tissues (*n* = 4). **l** WB analysis of the expressions of SLC7A11 and GPX4 in heart tissues (*n* = 4). *P < 0.05; **P < 0.01; ***P < 0.001; ****P < 0.0001. DIC doxorubicin-induced cardiomyopathy, Fer-1 Ferrostatin-1, Dox doxorubicin, 5-HETE, 5-hydroxyicosatetraenoic acid, HW/TL heart weight/tibia length, LVEF left ventricular ejection fraction, LVFS left ventricular fractional shortening, LDH lactic dehydrogenase, HE Hematoxylin and Eosin, GSH glutathione, MDA malondialdehyde, WB Western Blot.

To explore the role of ALOX5 in DIC, AAV9-cTnT-*Alox5* was administered into mice through tail vein injection to specifically overexpress *Alox5* in cardiomyocytes two weeks before Dox administration (Fig. 2a). RT-qPCR, WB, and IF analyses verified the efficiency of *Alox5* overexpression in heart tissues (Figure S2A–S2C). ELISA assay revealed that 5-HETE levels were elevated in the hearts of Dox-treated mice and were further increased by *Alox5* overexpression (Fig. 2b). Given the established role of ferroptosis in the pathological process of DIC [8] and our previous finding that its inhibition conferred protection against Dox-induced cardiac dysfunction [16], we next investigated whether ALOX5 modulates DIC by regulating ferroptosis. Mice were pretreated with the ferroptosis inhibitor Ferrostatin-1 (Fer-1) two weeks before Dox administration and continued throughout the experiment. Dox-treated mice exhibited reduced body weight and heart weight/tibia length (HW/TL) and impaired cardiac function, as evidenced by decreased left ventricular ejection fraction (LVEF) and left ventricular fractional shortening (LVFS) (Fig. 2c–g). In addition, Dox treatment caused a significant increase in lactic dehydrogenase (LDH) release and marked histopathological damages, including cardiomyocytes disarrangement and cytoplasmic vacuolization (Fig. 2h, i). However, Fer-1 treatment significantly ameliorated these Dox-induced abnormalities. Solute carrier family 7 member 11 (SLC7A11)-mediated cystine uptake supports GSH synthesis, which is utilized by GPX4 to eliminate lipid peroxides and suppress ferroptosis; disruption of this axis leads to lipid peroxide accumulation and ferroptosis initiation [17, 18]. Accordingly, cardiac GSH levels, protein expressions of SLC7A11 and GPX4, and the lipid peroxidation end product malondialdehyde (MDA) levels were determined. Dox treatment decreased cardiac GSH levels and the protein expressions of SLC7A11 and GPX4, while increasing MDA levels; these changes were significantly ameliorated by Fer-1 (Fig. 2j–l). Together, these data indicate that ferroptosis plays a crucial role in the pathological process of DIC, and inhibiting ferroptosis exerts cardioprotective effects against DIC.

Cardiac dysfunction was exacerbated in *Alox5*-overexpressed mice that received Dox treatment, as indicated by further decreased body weight and HW/TL, lower LVEF and LVFS, higher LDH release, and worsened histopathological damages (Fig. 2c–i). Moreover, cardiomyocyte-specific *Alox5* overexpression exacerbated Dox-caused ferroptosis in cardiac tissues, as evidenced by a more pronounced decrease in GSH levels and increase in MDA levels, along with lower expressions of SLC7A11 and GPX4 (Fig. 2j–l). However, these aggravated cardiac damages due to *Alox5* cardiomyocyte-specific overexpression were ameliorated by Fer-1 administration. Collectively, these in vivo results suggest that cardiomyocyte-specific overexpression of *Alox5* aggravates DIC by intensifying ferroptosis.

### Inhibiting ALOX5 ameliorates DIC by suppressing ferroptosis in vivo

**Fig. 3:**
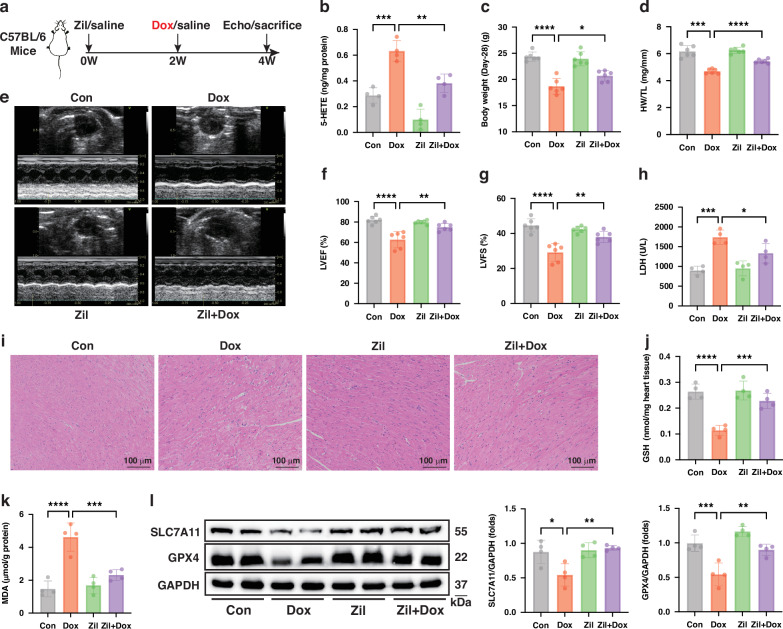
ALOX5 inhibition ameliorates DIC by suppressing ferroptosis in vivo. **a** Experimental design for investigating the effect of inhibiting ALOX5 on DIC in vivo. **b** 5-HETE content in murine heart tissues (*n* = 4). **c**, **d** Body weight and HW/TL of mice (*n* = 6). **e**–**g** Representative cardiac echocardiography images and quantitative analysis of LVEF and LVFS in mice (*n* = 6). **h** Serum LDH levels (*n* = 4). **i** Representative HE staining images of heart tissues (*n* = 3). **j**, **k** GSH content and MDA levels in heart tissues (*n* = 4). **l** WB analysis of the expressions of SLC7A11 and GPX4 in heart tissues (*n* = 4). *P < 0.05; **P < 0.01; ***P < 0.001; ****P < 0.0001. DIC doxorubicin-induced cardiomyopathy, Zil Zileuton, Dox doxorubicin, 5-HETE 5-hydroxyicosatetraenoic acid, HW/TL heart weight/tibia length, LVEF left ventricular ejection fraction, LVFS left ventricular fractional shortening; LDH lactic dehydrogenase, HE Hematoxylin and Eosin, GSH glutathione, MDA malondialdehyde, WB Western Blot.

To investigate the role of ALOX5 inhibition in DIC and evaluate its therapeutic potential, mice were pretreated with Zileuton (Zil), a well-established selective inhibitor of ALOX5, prior to Dox administration (Fig. 3a). Zil treatment effectively suppressed 5-HETE level in heart tissues (Fig. 3b) and significantly ameliorated Dox-induced body weight loss, HW/TL decrease, and cardiac dysfunction (Fig. 3c–g). Meanwhile, Zil alleviated the Dox-induced LDH release and histopathological damages (Fig. 3h, i). Furthermore, Zil treatment counteracted Dox-induced increase in cardiac MDA levels, and mitigated the downregulation of GSH levels and SLC7A11 and GPX4 expressions (Fig. 3j–l). The cardioprotective effect of Zil was further validated in an alternative DIC model established via tail vein injection of Dox (Figure S3). Collectively, these in vivo findings indicate that ALOX5 inhibition ameliorates DIC by inhibiting ferroptosis.

### *Alox5* overexpression aggravates DIC by promoting ferroptosis in vitro

To determine whether *Alox5* overexpression could aggravate Dox-induced cytotoxicity and ferroptosis in H9C2 cells, an *Alox5*-overexpressing plasmid was constructed. The efficiency of *Alox5* overexpression was confirmed using RT-qPCR and WB assays (Figure S4A, S4B). *Alox5* overexpression further increased the 5-HETE production in Dox-treated H9C2 cells and significantly enhanced Dox-induced cytotoxicity, as shown by increased LDH release, reduced GSH levels, and elevated MDA levels (Figure S4C–S4F). Additionally, *Alox5* overexpression augmented Dox-induced reactive oxygen species (ROS) accumulation and lipid peroxidation, as detected by the fluorescent probe Dihydroethidium (DHE) and BODIPY™ 581/591 C11 (Figure S4G, S4H). WB analysis further indicated that *Alox5* overexpression exacerbated the downregulation of SLC7A11 and GPX4 expressions induced by Dox (Figure S4I). In accordance with the in vivo results, Fer-1 treatment significantly mitigated Dox-induced cytotoxicity and counteracted the additional cytotoxic effects potentiated by *Alox5* overexpression (Figure S4D–S4I). Collectively, these data indicate that overexpression of *Alox5* aggravated Dox-induced cytotoxicity by promoting ferroptosis.

### ALOX5 inhibition and knockdown ameliorate DIC by suppressing ferroptosis in vitro

We next employed both pharmacological and genetic methods to inhibit and knockdown ALOX5 in H9C2 cells. Cells were incubated with Zil at different concentrations for 24 h. The CCK-8 assay confirmed that 50 μM Zil did not affect cell viability; therefore, this concentration was selected for the subsequent in vitro experiments (Figure S5A). Zil effectively suppressed the generation of 5-HETE in H9C2 cells (Figure S5B) and significantly ameliorated Dox-induced cytotoxicity, as evidenced by increased cell viability and reduced LDH release (Figure S5C and S5D). Additionally, Zil administration alleviated Dox-induced GSH level decrease, MDA level elevation, and intracellular ROS and lipid peroxidation increase in cells (Figure S5E–S5H). WB analysis indicated that the downregulated expressions of SLC7A11 and GPX4 in Dox-treated cells were also attenuated by Zil intervention (Figure S5I).

We also used siRNA to knockdown *Alox5* in H9C2 cells. The efficiency of *Alox5* knockdown was verified by RT-qPCR, WB, and ELISA assays (Figure S6A–S6C). Knockdown of *Alox5* in H9C2 cells improved cell viability reduction and alleviated LDH release caused by Dox (Figure S6D and S6E). In addition, *Alox5* knockdown elevated the intracellular GSH level, reduced the MDA level, decreased intracellular ROS and lipid peroxidation, and restored the expressions of SLC7A11 and GPX4 in Dox-treated H9C2 cells (Figure S6F–S6J). Together with the pharmacological inhibition results, these findings suggest that both ALOX5 inhibition and knockdown ameliorate Dox-induced cytotoxicity through suppressing ferroptosis.

### Upregulation of ALOX5 in DIC is transcriptionally regulated by EGR1

The mechanism underlying Dox-induced ALOX5 upregulation was further explored. The concurrent upregulation of ALOX5 mRNA and protein levels prompted us to hypothesize that it might be mediated by transcriptional regulation. Based on a previous study reporting four candidate transcription factors that could bind to the *Alox5* promoter region, including E2F transcription factor 1 (E2F1), early growth response 1 (EGR1), specificity protein 1 (SP1), and YY1 [19], we analysed their expressions in the sequencing data from NRCMs treated with Dox or DMSO for either 6 or 24 h. Among these four transcription factors, the expression patterns of *E2f1* and *Egr1* were consistent with that of *Alox5* (Figure S7A). Subsequent RT‑qPCR and WB analyses confirmed that the mRNA and protein levels of E2F1 and EGR1 were elevated after Dox treatment (Figure S7B and S7C). Subsequently, the effects of *E2f1* or *Egr1* knockdown on the expression of ALOX5 in H9C2 cells were explored (Figure S7D–S7G). Both RT-qPCR and WB results revealed that *Egr1* knockdown significantly suppressed Dox-induced upregulation of the mRNA and protein levels of ALOX5, whereas *E2f1* knockdown exerted no significant effect on ALOX5 expression (Figure S7H–S7K). These results suggest that EGR1 acts as an upstream transcriptional regulator of ALOX5 in DIC.

### Upregulation of the ALOX5 metabolism axis contributes to Dox-induced cardiomyocyte ferroptosis

**Fig. 4:**
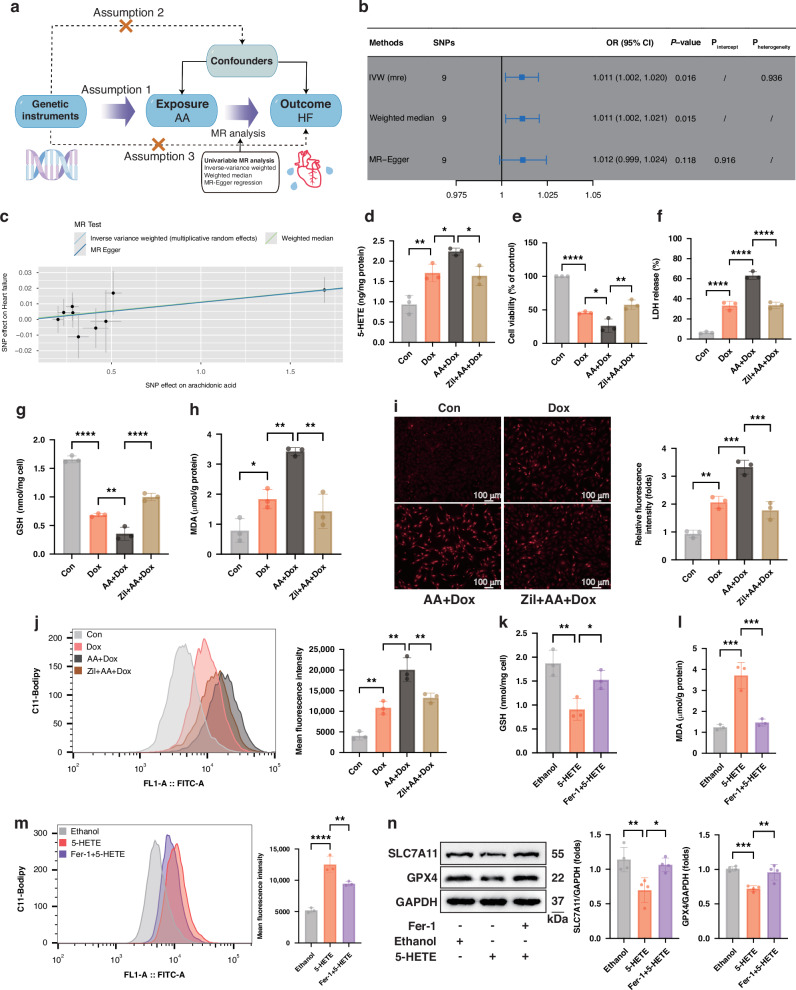
Upregulation of the ALOX5 metabolism axis contributes to Dox-induced cardiomyocyte ferroptosis. **a** The schematic diagram of the Mendelian randomization study design, which is based on three assumptions: (1) The IVs should be robustly associated with the exposure (AA). (2) IVs should not be associated with any confounding factors. (3) IVs exert effects on the outcome (HF) only through exposure and not through other pathways. **b** Mendelian randomization analysis of the association between plasma AA level and HF. **c** Scatterplots of the effect size for each SNP on AA and HF risk. **d** 5-HETE content in H9C2 cells (*n* = 3). **e** CCK8 analysis of the cell viability (*n* = 3). **f**–**h** LDH release, GSH content, and MDA levels in H9C2 cells (*n* = 3). **i** DHE staining of H9C2 cells (*n* = 3). **j** Flow cytometer analysis with BODIPY™ 581/591 C11 staining (*n* = 3). **k**, **l** GSH content and MDA levels in H9C2 cells (*n* = 3). **m** Flow cytometer analysis with BODIPY™ 581/591 C11 staining (*n* = 3). **n** WB analysis of the expressions of SLC7A11 and GPX4 in H9C2 cells (*n* = 4). *P < 0.05; **P < 0.01; ***P < 0.001; ****P < 0.0001. Dox doxorubicin, IVs instrumental variables, AA arachidonic acid, HF heart failure, SNP single-nucleotide polymorphisms, OR odds ratio, Zil Zileuton, LDH lactic dehydrogenase, GSH glutathione, MDA malondialdehyde, DHE Dihydroethidium, 5-HETE 5-hydroxyicosatetraenoic acid, Fer-1 Ferrostatin-1, WB Western Blot.

Mendelian randomization (MR) analysis was performed to investigate the causal association between ALOX5 substrate (AA) and HF (Fig. 4a). Results indicated that higher plasma level of AA was positively associated with an increased risk of HF (odds ratio = 1.011, 95% confidence interval: 1.002–1.020, *P* = 0.016; Fig. 4b, c). No evidence of horizontal pleiotropy or heterogeneity was observed (Fig. 4b). Subsequently, to investigate whether ALOX5 contributes to Dox-induced cardiomyocyte ferroptosis through the metabolism of polyunsaturated fatty acid (PUFA) and the production of lipid peroxides, a PUFA-enriched model was established by incubating H9C2 cells with AA for 24 h. AA supplementation significantly elevated the level of 5-HETE in Dox-treated cells (Fig. 4d). Compared to Dox treatment alone, Dox combined with AA supplementation resulted in more severe cytotoxic and ferroptotic responses, as evidenced by decreased cell viability, increased LDH release, decreased GSH content, increased MDA level, and enhanced ROS production and lipid peroxidation (Fig. 4e–j). Importantly, these aggravated effects were attenuated by ALOX5 inhibition with Zil, suggesting that ALOX5-mediated phospholipid peroxidation plays a pivotal role in Dox-induced cytotoxicity and cardiomyocyte ferroptosis.

To further elucidate whether ALOX5-derived 5-HETE directly triggers ferroptosis, H9C2 cells were treated with 5-HETE. Notably, 5-HETE incubation decreased the GSH content and increased the MDA levels (Fig. 4k, l). Moreover, 5-HETE elevated the intracellular lipid peroxidation levels and downregulated the expressions of SLC7A11 and GPX4 (Fig. 4m, n). In addition, these pro-ferroptotic effects of 5-HETE were significantly mitigated by Fer-1.

Taken together, these results suggest that the upregulation of ALOX5 metabolism of AA to 5-HETE contributes to Dox-induced ferroptosis in cardiomyocytes.

### The regulatory effects of the ALOX5 metabolism axis on ferroptosis are NRF2-dependent

**Fig. 5:**
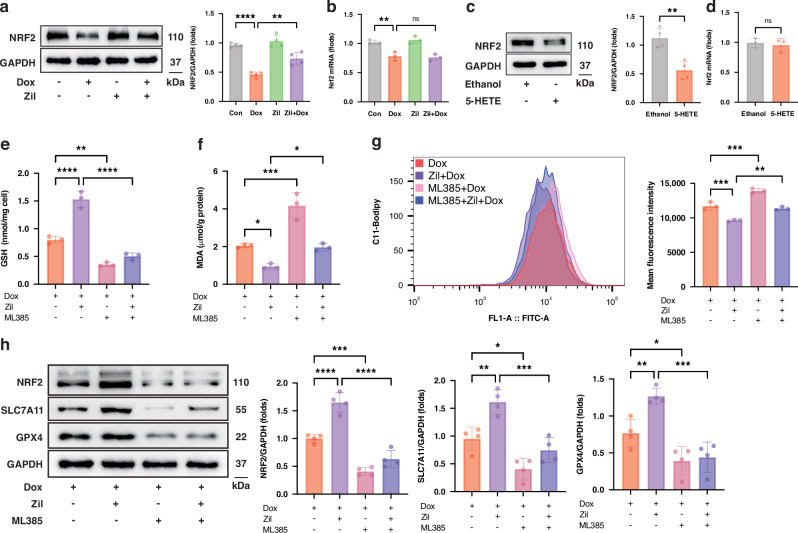
ALOX5 metabolism axis regulates ferroptosis in an NRF2-dependent manner. **a**, **c** WB analysis of NRF2 expression in H9C2 cells (*n* = 4). **b**, **d** *Nrf2* mRNA level in H9C2 cells (*n* = 3). **e**, **f** GSH content and MDA levels in H9C2 cells (*n* = 3). **g** Flow cytometer analysis with BODIPY™ 581/591 C11 staining (*n* = 3). **h** WB analysis of the expressions of NRF2, SLC7A11, and GPX4 in H9C2 cells (*n* = 4). ns no significance; *P < 0.05, **P < 0.01, ***P < 0.001, ****P < 0.0001. WB Western Blot, Dox doxorubicin, Zil Zileuton, 5-HETE 5-hydroxyicosatetraenoic acid, GSH glutathione, MDA malondialdehyde.

Studies have reported that NRF2 binds to the antioxidant response element to promote the transcription of SLC7A11 and GPX4, thereby protecting cardiomyocytes from ferroptotic damage [7, 20]. Notably, inhibiting ALOX5 was found to reduce ischemia-reperfusion (I/R) injury by counteracting the I/R-induced downregulation of NRF2 [11]. To investigate the mechanism underlying the regulatory effects of the AA/ALOX5/5-HETE axis on the expressions of SLC7A11 and GPX4, the effects of ALOX5 manipulation and 5-HETE treatment on NRF2 levels were assessed. Dox treatment significantly decreased NRF2 protein levels both in vivo and in vitro, while ALOX5 inhibition or knockdown restored them (Fig. 5a, Figure S8A, S8B). Conversely, *Alox5* overexpression further downregulated the protein level of NRF2 (Figure S8C, S8D). However, no significant change was observed in the mRNA level of *Nrf2* by ALOX5 inhibition (Fig. 5b). Additionally, 5-HETE decreased the protein level of NRF2 without affecting its mRNA level (Fig. 5c, d). These findings suggest that ALOX5 and 5-HETE modulate the expression of NRF2 at the protein level.

Furthermore, to explore whether ALOX5 inhibition mitigates Dox-induced ferroptosis by modulating NRF2, H9C2 cells were treated with ML385, a specific inhibitor of NRF2. The results revealed that inhibiting NRF2 with ML385 counteracted the protective effects of ALOX5 inhibition against Dox-induced ferroptosis in H9C2 cells. Specifically, ML385 enhanced Dox-induced ferroptotic damage, as evidenced by lower GSH content, higher MDA level and lipid peroxidation, and lower expressions of SLC7A11 and GPX4 (Fig. 5e–h). Meanwhile, ML385 abrogated the upregulation of GSH content and downregulation of MDA level and lipid peroxidation caused by ALOX5 inhibition (Fig. 5e–g). The effect of ALOX5 inhibition in upregulating the expressions of SLC7A11 and GPX4 was also blocked by ML385 (Fig. 5h). These findings suggest that the ALOX5 metabolic pathway modulates Dox-induced cardiomyocyte ferroptosis in an NRF2-dependent manner.

### ALOX5 metabolism axis facilitates NRF2 ubiquitination-dependent degradation via the PI3K/AKT/GSK-3β pathway in DIC

**Fig. 6:**
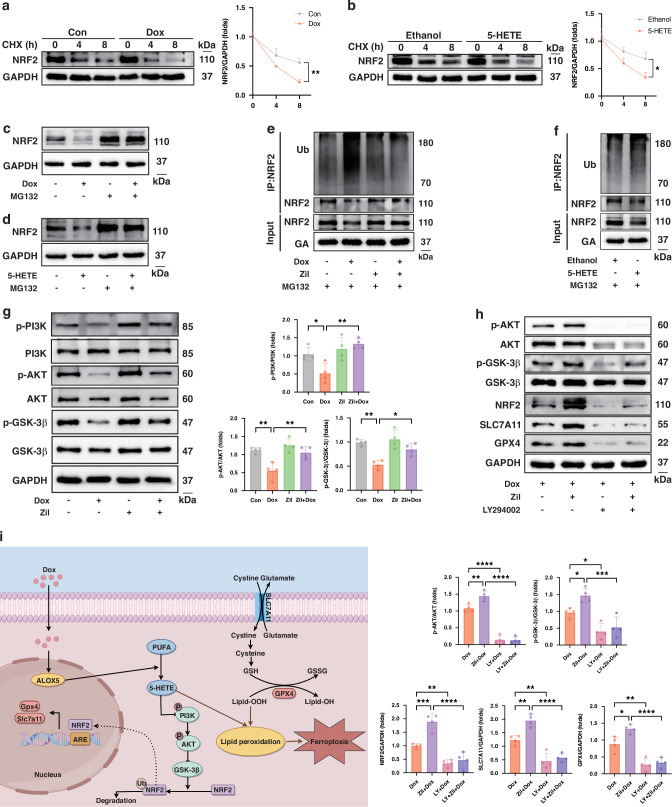
ALOX5 metabolism axis facilitates NRF2 ubiquitination-dependent degradation via the PI3K/AKT/GSK-3β pathway. **a**, **b** WB analysis of NRF2 protein levels in H9C2 cells with CHX (100 μg/ml) incubation at different times (*n* = 3). **c**, **d** Protein levels of NRF2 in Dox or 5-HETE-treated H9C2 cells with or without MG132 (10 μM) treatment for 6 h (*n* = 3). **e** Immunoprecipitation assay of cell lysates from Dox-treated H9C2 cells exposed to MG132 with or without Zil treatment, precipitated with anti-NRF2 antibody and then immunoblotted with anti-ubiquitin antibody (*n* = 3). **f** Immunoprecipitation assay of cell lysates from ethanol or 5-HETE-treated H9C2 cells exposed to MG132, precipitated with anti-NRF2 antibody and then immunoblotted with anti-ubiquitin antibody (*n* = 3). **g** WB analysis of protein levels of p-PI3K, PI3K, p-AKT, AKT, p-GSK-3β, and GSK-3β (*n* = 4). **h** WB analysis of protein levels of p-AKT, AKT, p-GSK-3β, GSK-3β, NRF2, SLC7A11, and GPX4 (*n* = 4). **i** Upregulation of the ALOX5 metabolism axis promotes cardiomyocyte ferroptosis in DIC via PI3K/AKT/GSK-3β mediated NRF2 ubiquitination-dependent degradation. *P < 0.05; **P < 0.01; ***P < 0.001; ****P < 0.0001. WB Western Blot, CHX cycloheximide, Dox doxorubicin, 5-HETE 5-hydroxyicosatetraenoic acid, Zil Zileuton, LY LY294002, DIC doxorubicin-induced cardiomyopathy.

Considering that the ALOX5 metabolism axis modulates NRF2 at the protein level, the protein stability of NRF2 was evaluated. Using the protein synthesis inhibitor cycloheximide (CHX), we found that both Dox and 5-HETE treatment significantly accelerated NRF2 protein degradation (Fig. 6a, b). The proteasome inhibitor MG132 could counteract the downregulation effects of Dox and 5-HETE on NRF2 protein levels (Fig. 6c, d). In addition, the ubiquitination level of NRF2 was significantly upregulated in Dox and 5-HETE-treated cells, whereas the inhibition of ALOX5 decreased the NRF2 ubiquitination level (Fig. 6e, f).

To identify the mechanism by which the ALOX5 metabolic pathway modulates the ubiquitination of NRF2 in DIC, Kyoto Encyclopedia of Genes and Genomes (KEGG) analysis was conducted using DEGs from NRCMs treated with Dox or DMSO for 24 h, which revealed significant enrichment of the PI3K/AKT signaling pathway in DIC (Figure S9A). Since previous studies have reported that the PI3K/AKT pathway could regulate NRF2 stability through GSK-3β-mediated NRF2 ubiquitination [21], we detected the level of PI3K/AKT/GSK-3β and their phosphorylated levels in Dox and 5-HETE-treated H9C2 cells. The ratio of phosphorylated PI3K (p-PI3K) to total PI3K, phosphorylated AKT (p-AKT) to total AKT, and phosphorylated GSK-3β (p-GSK-3β) to total GSK-3β were decreased in Dox-treated cells, suggesting the suppression of PI3K/AKT signaling and consequent activation of GSK-3β in DIC (Fig. 6g). However, these effects were significantly reversed by ALOX5 inhibition. Meanwhile, 5-HETE treatment also inhibited PI3K/AKT signaling and activated GSK-3β (Figure S9B).

To validate whether ALOX5 inhibition stabilizes NRF2 via the PI3K/AKT pathway, H9C2 cells were treated with the PI3K inhibitor LY294002. Notably, LY294002 diminished the protective effects of ALOX5 inhibition against DIC and ferroptotic damage (Figure S10A–S10F). In addition, the effects of ALOX5 inhibition on the inactivation of GSK-3β, downregulation of NRF2 ubiquitination, and upregulation of NRF2, SLC7A11, and GPX4 protein levels, were abrogated by LY294002 (Fig. 6h and S10G). These results reveal that the ALOX5 metabolism axis facilitates the ubiquitination-dependent degradation of NRF2 via the PI3K/AKT/GSK-3β pathway in DIC.

### ALOX5 inhibition ameliorates DIC without compromising the antitumor efficiency of Dox

Finally, to determine whether ALOX5 inhibition interferes with the chemotherapeutic efficiency of Dox, 1 μM Dox was administered alongside various concentrations of Zil to MDA-MB-231 and HepG2 tumor cell lines. As expected, Dox significantly reduced the viability of both cell lines (Figure S11A and S11B). However, Zil did not diminish the antitumor effect of Dox at any concentration tested. These results indicate that ALOX5 inhibition protects against DIC while preserving the antitumor efficacy of Dox.

## Discussion

The present study revealed that ALOX5 was markedly upregulated in both in vivo and in vitro models of DIC. *Alox5* overexpression exacerbated DIC, while ALOX5 inhibition and knockdown alleviated it by modulating cardiomyocyte ferroptosis. Mechanistically, the ALOX5-mediated metabolism of AA to 5-HETE promotes Dox-induced ferroptosis. Notably, the ALOX5 metabolism axis facilitates the ubiquitination-dependent degradation of NRF2 in DIC through PI3K/AKT/GSK-3β signaling, leading to subsequent downregulation of SLC7A11 and GPX4 (Fig. 6i).

Accumulating evidence has implicated that ALOX5 plays a pivotal role in the development of various cardiovascular diseases. Xie et al. demonstrated that ALOX5 deficiency ameliorated transverse aortic constriction-induced cardiac hypertrophy [10]. In addition, inhibiting ALOX5 with MK886 significantly improved I/R-mediated cardiac contractile dysfunction and decreased apoptosis and oxidative stress [11]. Moreover, ALOX5 silencing reduced LPS-induced apoptosis, inflammation, and ferroptosis in AC16 cells [22]. In the present study, we identified ALOX5 as a key contributor to DIC pathogenesis. We observed that Dox upregulates ALOX5, which aggravates DIC upon overexpression. Conversely, both pharmacological inhibition and genetic knockdown of ALOX5 alleviated DIC, confirming its pivotal pathogenic role.

Dox challenge is known to trigger ferroptotic phenotypes in cardiomyocytes, and targeting ferroptosis was found to confer protective effects against Dox-induced cardiac dysfunction [23–25]. Given the predominant role of ferroptosis in DIC compared to other forms of cell death, it represents a highly promising preventative therapeutic target for DIC [8]. Recent studies have depicted a close relationship between ALOX5 and ferroptosis. For instance, elevated ALOX5 was found to promote the occurrence of autophagy-dependent ferroptosis in melanoma by activating the AMPK/mTOR pathway and downregulating GPX4 expression [15]. Similarly, inactivation of the ALOX5 gene could abrogate the ferroptosis activity in the striatal neurons of mice with Huntington’s disease [13]. In alignment with these findings, the present study specifically demonstrates that ALOX5 plays a modulatory role in ferroptosis within the context of DIC. Our results indicated that *Alox5* overexpression aggravated Dox-induced cardiomyocyte ferroptosis, whereas both pharmacological inhibition and genetic knockdown of ALOX5 attenuated this effect.

The oxidation of PUFAs is essential for the initiation of ferroptosis in response to a variety of stimuli [26]. Notably, LOXs play a crucial role in ferroptosis by generating initial lipid peroxides [27, 28]. Thus, this study hypothesized that ALOX5-mediated metabolism of PUFA and subsequent generation of lipid peroxides might be a critical mechanism underlying Dox-induced ferroptosis. AA, an omega-6 PUFA and a key component of cell membrane phospholipids, serves as the primary metabolic substrate of ALOX5 [29]. Nonetheless, observational findings on AA and cardiovascular diseases remain controversial, and moreover, randomized controlled trials investigating AA supplementation are limited [30–32]. The present study established a PUFA-enriched model with AA supplementation, which was found to aggravate Dox-induced cardiomyocyte ferroptosis. Importantly, this detrimental effect was mitigated by ALOX5 inhibition, further substantiating the essential role of ALOX5-mediated AA metabolism in DIC. This finding suggests that the supplementation of PUFAs, especially AA, in chemotherapy patients warrants careful consideration. Beyond AA, our focus also extended to 5-HETE, an ALOX5-derived oxidative metabolite of AA. Previous studies have implicated the role of 5-HETE in promoting lipid peroxidation and ferroptosis. For instance, 5-HETE treatment was shown to diminish the protective effects conferred by ALDH2 against Aβ-induced lipid peroxidation; in addition, suppressing erastin-induced 5-HETE production has been shown to inhibit ferroptosis induced by ACSL4 overexpression [33, 34]. In line with these studies, we observed that 5-HETE level was significantly elevated in DIC, which was further amplified by AA supplementation. Furthermore, 5-HETE stimulation directly triggered ferroptotic damage in cardiomyocytes, indicating that the ALOX5-mediated AA metabolism axis constitutes a critical initiating step in the development of Dox-induced ferroptosis.

In recent years, the transcription factor NRF2 has been recognized as an important mitigator of ferroptosis, regulating a multitude of downstream target genes involved in iron metabolism, intermediate metabolism, and the synthesis and metabolism of glutathione [35, 36]. This study revealed that both Dox and 5-HETE treatment led to a downregulated NRF2 protein level, while ALOX5 inhibition or knockdown upregulated NRF2 and conferred cardioprotective effects against DIC. The activity of NRF2 is controlled by a broad range of transcriptional regulators and post-translational modifications. Given that the ALOX5 metabolic axis did not significantly alter the mRNA level of *Nrf2*, we explored its impact on protein stability. The stability of NRF2 is predominantly regulated by three different E3-ubiquitin ligase complexes: Kelch-like ECH-associated protein 1-Cullin 3-Ring box 1, S-phase kinase-associated protein 1-Cullin1-Rbx1/β-transducin repeat-containing protein (β-TRCP), and synoviolin/Hrd1 [35]. The present study confirmed that both Dox and 5-HETE facilitated the ubiquitination-dependent degradation of NRF2. Meanwhile, KEGG analysis indicated that the PI3K/AKT pathway was enriched in DIC, and treatment with Dox and 5-HETE effectively inhibited the PI3K/AKT signaling, while activated GSK-3β. Notably, the PI3K/AKT signaling pathway has been shown to play an essential role in DIC, with PI3K/AKT/GSK-3β-mediated NRF2 ubiquitin/proteasome degradation being implicated in ferroptosis [21, 37–41]. GSK-3β functions as a negative regulator of NRF2, which phosphorylates NRF2 and creates a recognition motif for the E3 ligase adapter β-TrCP, leading to the ubiquitination and subsequent proteasome degradation of NRF2 [42]. This study demonstrated that inhibiting PI3K/AKT activation with LY294002 abrogated the protective effects conferred by ALOX5 inhibition against Dox-induced ferroptotic damage and ubiquitination-dependent degradation of NRF2. Therefore, the ALOX5 metabolism axis regulates the ubiquitination-dependent degradation of NRF2 in DIC through a PI3K/AKT/GSK-3β signaling-dependent manner.

Strategies currently employed to alleviate DIC primarily include adjustments to chemotherapy drug delivery and dosage, as well as the co-administration of cardioprotective medications. However, these strategies are often hindered by the presence of side effects, poor clinical feasibility, or a reduction in the anti-tumor efficacy of Dox. Notably, ALOX5 represents a viable therapeutic target within the AA/ALOX5/5-HETE axis. Zil is the only approved ALOX5 inhibitor, which has been applied in the clinical treatment of asthma [43]. The present study employed Zil to pharmacologically inhibit ALOX5, revealing that Zil did not compromise the antitumor efficiency of Dox in various cancer cells. These findings not only highlight that the ALOX5 metabolism axis represents a feasible treatment strategy for DIC but also suggest a potential new clinical application for Zil.

## Conclusions

Our study demonstrates that upregulation of the ALOX5 metabolism axis promotes cardiomyocyte ferroptosis in DIC via PI3K/AKT/GSK-3β-mediated NRF2 ubiquitination-dependent degradation. These findings not only highlight the ALOX5 metabolism axis as a promising therapeutic target for DIC, but also suggest the potential for expanding the therapeutic applications of Zil.

## Supplementary information

Supplementary materials

Supplementary materials-Original Western Blot images

## Data Availability

All original data used for this study are available from the corresponding author upon reasonable request.
