# Supplementary material for: Arachidonate lipoxygenase 5 metabolism axis promoting ferroptosis: a potential druggable target for doxorubicin-induced cardiomyopathy
Source: Br J Cancer. 2026 Apr 6;134(11):1529–40. doi: 10.1038/s41416-026-03376-3 (PMC13183924; doi:10.1038/s41416-026-03376-3)
Supplement: Supplementary file 1 — Supplementary materials [file 41416_2026_3376_MOESM1_ESM.docx]

**Supplementary Materials for**

**Arachidonate Lipoxygenase 5 Metabolism Axis Promoting Ferroptosis: a Potential Druggable Target for Doxorubicin-Induced Cardiomyopathy**

Lu Chen ^1, 2, 5^, Xingang Sun ^3, 5^, Han Zhang ^1^, Xuan Zhang ^1^, Mairedan Muhetarijiang ^1^, Zuoshi Wen ^1^, Chenxi Li ^1^, Mengjia Chen ^1^, Zhangquan Ying ^1^, Shuai Yuan ^4^, Liangrong Zheng ^1, *^, Ting Chen ^1, *^

^1^ Department of Cardiology, The First Affiliated Hospital, School of Medicine, Zhejiang University, 79 Qingchun Road, Hangzhou, Zhejiang Province, China

^2^ Department of Critical Care Medicine, Sir Run Run Shaw Hospital, School of Medicine, Zhejiang University, Hangzhou, Zhejiang Province, China

^3^ Department of Cardiology, Zhejiang Provincial People's Hospital, People's Hospital of Hangzhou Medical College, 158 Shangtang Road, Hangzhou, Zhejiang Province, China

^4^ Department of Echocardiography and Vascular Ultrasound Center, The First Affiliated Hospital, School of Medicine, Zhejiang University, 79 Qingchun Road, Hangzhou, Zhejiang Province, China

^5^ These authors contributed equally: Lu Chen, Xingang Sun.

^*^: Corresponding author: Ting Chen, E-mail address: ct010151452@zju.edu.cn and Liangrong Zheng, E-mail address: 1191066@zju.edu.cn.

**This file includes:**

**Supplementary Methods**

**Supplementary Tables S1 to S5**

**Supplementary Figures S1 to S11**

**Supplementary Methods**

**RNA sequencing analysis**

The expression profiling data utilized in this study were sourced from the Gene Expression Omnibus (GEO) database (https://www.ncbi.nlm.nih.gov/geo/). The “GEOquery” package in R ^1^ was employed to retrieve the raw datasets (GSE166957). Differentially expressed genes (DEGs) were identified using the “limma” R package ^2^ , with a stringent threshold of *P* < 0.05 and an absolute log2 fold change (FC) > 2. Additionally, a set of 484 ferroptosis-related genes (FRGs) was retrieved from the FerrDb database (<http://www.zhounan.org/ferrdb/current/>; accessed: January 2023). Differentially expressed ferroptosis-related genes (DEFRGs) were identified by intersecting DEGs with FRGs, and their expression patterns were visualized using volcano plots. To further elucidate the biological functions associated with the DEGs, Kyoto Encyclopedia of Genes and Genomes (KEGG) pathway analysis was performed in R using the “clusterProfiler” package ^3^.

**Human heart samples**

Left ventricular tissues were collected from 3 healthy donors and 3 heart failure (HF) patients (left ventricular ejection fraction [LVEF] < 40%) undergoing heart transplantation. Human samples were collected according to the human research protocol, approved by the Research Ethics Committees of the First Affiliated Hospital of Zhejiang University (Approval Reference No. 2021/330). Written informed consents were obtained from all patients in accordance with the Declaration of Helsinki. Clinical details of the HF patients were shown in Table S1.

**Animals and treatment**

8-10 weeks male C57BL/6 mice weighing 20-25g were obtained from the Academy of Medical Science (Zhejiang, China) and housed in a thermostatically regulated environment (22 ± 2°C) with 12-hour light/dark cycles. Mice were stratified by body weight and randomly assigned to experimental groups using a computer-generated sequence to ensure comparable initial weight across groups. All animal procedures received ethical approval from the Animal Experimental Ethical Inspection of the First Affiliated Hospital, Zhejiang University, School of Medicine, and were strictly followed by the relevant ethical standards (Approval Number: 20241290) and based on the Guide for the Care and Use of Laboratory Animals published by the US National Institutes of Health (NIH).

The doxorubicin-induced cardiomyopathy (DIC) model was established through intraperitoneal injection of doxorubicin (Dox) at a total dosage of 24 mg/kg over two weeks (3 times weekly) and through tail vein injection of Dox at a total dosage of 20 mg/kg over four weeks (1 time weekly)^4^. To investigate the role of cardiomyocyte-specific *Alox5* overexpression in DIC, mice were randomly assigned to the following 6 experimental groups (n = 5): adeno-associated virus (AAV)-NC, AAV-NC+Dox, AAV-NC+Dox+Ferrostatin-1 (Fer-1), AAV-*Alox5*, AAV-*Alox5*+Dox, and AAV-*Alox5*+Dox+Fer-1. Mice were injected with either AAV9-cTnT-NC or AAV9-cTnT-*Alox5* (HANBIO, China) through the tail vein two weeks before Dox treatment. Meanwhile, mice received daily intraperitoneal injections of the ferroptosis inhibitor Fer-1 (1 mg/kg) or an equal volume of solvent started two weeks before Dox treatment and continued throughout the experiment ^5^. After a subsequent two-week period, mice were intraperitoneally injected with Dox to establish the DIC model.

To investigate the effect of ALOX5 inhibition in DIC, mice were randomly assigned to the following 4 groups (n = 6): Con, Dox, Zileuton (Zil), and Zil+Dox. Before the Dox treatment, mice were pre-treated with the ALOX5 selective inhibitor Zil (100 mg/kg, daily) or an equal volume of solvent for two weeks orally and maintained throughout the experiment ^6^. After a subsequent two-week period, the DIC model was established through intraperitoneal or tail vein injection of Dox.

Twenty-four hours after the final injection of Dox, the transthoracic echocardiography assessment (GE Vivid E95 ultrasound system, General Electric Company) was conducted under anesthesia induced by intraperitoneal injection of a single dose of 1% sodium pentobarbital. The mice were maintained normothermic throughout the entire echocardiographic image acquisition process with a temperature-controlled heating pad. The technician performing the echocardiographic examinations was only aware of the random codes and was blinded to the group allocation. LVEF (%) and left ventricular fractional shortening (LVFS, %) were measured and calculated. The researcher responsible for analyzing the ultrasound images (calculating LVEF% and LVFS%) was also provided with data files identified only by the random codes and was blinded to the group information. Subsequently, the mice were euthanized via cervical dislocation under deep anesthesia. The cardiac tissues were harvested and fixed in a 4% paraformaldehyde solution for subsequent histological analysis or frozen in liquid nitrogen for preservation. The serum was extracted from the collected blood samples and preserved at -80 °C for subsequent studies. Information on the reagents used in this study was shown in Table S2.

**Cell culture and treatment**

The H9C2 cells were bought from the Cell Bank of the Chinese Academy of Sciences. The cells were routinely maintained in DMEM, enriched with 10% fetal bovine serum and 1% penicillin/streptomycin solution at 37°C under a 5% CO_2_ atmosphere.

To establish the DIC model in vitro, H9C2 cells were treated with 1 μM Dox for 24 hours. In the subsequent rescue experiments, the cells were pretreated with 50 μM Zil or 10 μM Fer-1 24 hours before Dox administration and maintained throughout the experiment ^5,7^. Furthermore, to investigate the role of ALOX5 metabolism axis in DIC, 50 μM arachidonic acid (AA) and 5 μM 5-hydroxyicosatetraenoic acid (5-HETE) were applied to incubate H9C2 cells for 24 hours ^8^. In addition, 10 μM LY294002 was used to inhibit PI3K/AKT, and 5 μM ML385 was used to inhibit NRF2 in H9C2 cells ^9,10^.

**Histological analysis**

Myocardial tissue samples of mice were immersed in 4% paraformaldehyde for 24 hours to ensure proper fixation and then embedded in paraffin. The embedded tissues were sectioned into 5 μm slices. Hematoxylin and Eosin (H&E) staining was conducted to assess the histological alterations. Immunohistochemistry (IHC) staining was performed to measure the protein expression level of ALOX5. The images were captured utilizing a light microscope (Olympus, BX53, Japan) and analyzed using the Image J software. The antibodies used in IHC analysis were detailed in Table S3.

**Immunofluorescence (IF) staining**

The myocardial tissue samples were deparaffinized, rehydrated, and blocked with bovine serum albumin. The primary antibodies against ALOX5 and cardiac Troponin-T were used to incubate the samples at 4°C overnight. The H9C2 cells were fixed in 4% paraformaldehyde and then permeabilized with 0.5% Triton X-100 at room temperature. After blocking with BSA, the cells were incubated with the anti-ALOX5 antibody at 4°C overnight. The next day, the heart tissues and cells were incubated with the secondary Cy3-conjugated and 488-conjugated antibodies for 1 hour at room temperature, and nuclei were counterstained with DAPI for 15 minutes at room temperature. The images were captured using the confocal microscope (Olympus FV3000, Japan) and analyzed using the Image J software. The antibodies used in IF staining were detailed in Table S3.

**Enzyme-linked immunosorbent assay (ELISA)**

The heart tissues and H9C2 cells were lysed using cell lysis buffer, and the concentration of 5-HETE was determined using the 5-HETE ELISA Kit, following the protocols provided by the manufacturer. The protein content was quantified using the enhanced BCA protein assay kit.

**Cell viability**

H9C2 cells were seeded into 96-well plates and the cell viability was evaluated using the CCK-8 kit at the end of treatments. 10 μL CCK-8 solution was added to 90 μL fresh medium in each well and incubated for 2 hours in the cell culture incubator. Thereafter, the absorbance at 450 nm was detected with a microplate reader (SpectraMax i3x, USA)

**Cell transfection with plasmids and siRNAs**

Gene overexpression and knockdown in H9C2 cells were performed using Lipofectamine 3000 reagent according to the manufacturer’s protocol. To overexpress *Alox5*, pCMV-*Alox5*(rat)-3×Flag-Neo plasmid (Miaoling, China) was transfected into H9C2 cells, with the pCMV-T7-MCS-3×FLAG-WPRE-Neo plasmid as negative control. *Alox5* siRNA (5’-GCAAGAGGACCTCATGTTT-3’) (RiboBio, China) was transfected to H9C2 cells to knockdown *Alox5*, with the scrambled siRNA (5’-GGCTCTAGAAAAGCCTATGC-3’) as the negative control. Additionally, *Egr1* siRNA (5’-GGACUUAAAGGCUCUUAAUTT-3’) and *E2f1* siRNA (5’-CUGUUAGGCCUGGAGCAAGAATT-3’) (HANBIO, China) were transfected into H9C2 cells to knockdown *Egr1* and *E2f1*, with the scrambled siRNA (5’-UUCUCCGAACGUGUCACGUTT-3’) as the negative control.

**Lactic dehydrogenase (LDH) detection**

The LDH levels in the serum extracted from mice blood samples were detected using the LDH assay kit. Additionally, the LDH levels in the supernatant of the H9C2 cells were ascertained with the LDH Release Assay Kit according to the manufacturer's instructions. The corresponding absorbances were measured using a microplate reader (SpectraMax i3x, USA).

**Glutathione (GSH) and malondialdehyde (MDA) content detection**

The heart tissues and cells were lysed and then employed to assess GSH content and MDA levels with the GSH and GSSG Assay Kit and the Lipid Peroxidation MDA Assay Kit, following the manufacturer's instructions. The absorbances at 412 nm for GSH content detection and 532 nm for MDA levels detection were measured using a microplate reader (SpectraMax i3x, USA).

**Lipid peroxidation and reactive oxygen species (ROS) detection**

To quantify lipid peroxidation, cells were washed with Hank’s balanced salt solution (HBSS) and then incubated with 5 μM C11-Bodipy (BODIPY™ 581/591 C11) at 37°C in the dark for 30 minutes. Subsequently, the cells were treated with trypsin and resuspended in HBSS. The fluorescence intensity was then determined using a flow cytometer (BECKMAN CytoFLEX, USA). To detect intracellular ROS, cells were washed with HBSS and then incubated with 10 μM DHE probe at 37°C in the dark for 30 minutes. The images were captured using the microscope (Olympus IX 73, Japan), and the average fluorescence intensity was measured using Image J software.

**RNA extraction and real-time quantitative PCR (RT-qPCR)**

Total RNA was extracted from the heart tissues and H9C2 cells with Trizol reagent, following the manufacturer’s instructions. Subsequently, cDNA was generated from 1 µg of the extracted RNA in a 20 µL reaction volume using the cDNA Synthesis Kit. The synthesized cDNA was then subjected to RT-qPCR using the Low ROX SYBR Green qPCR Master Mix. The primer sequences employed were detailed in Table S4.

**Western Blot (WB)**

Total proteins were isolated from the heart tissues and H9C2 cells using RIPA lysis buffer supplemented with protease and phosphatase inhibitors. Subsequently, equivalent quantities of these proteins were separated by sodium dodecyl sulfate-polyacrylamide gel electrophoresis (SDS-PAGE) and transferred to 0.22 μm PVDF membranes. The membranes were then blocked with 5% skim milk solution in Tris-buffered saline for 1 hour at room temperature. Subsequently, the blots were subjected to an overnight incubation with their respective primary antibodies at 4°C. The primary antibodies included: ALOX5, GAPDH, solute carrier family 7 member 11 (SLC7A11), glutathione peroxidase 4 (GPX4), E2F1, EGR1, NRF2, p-PI3K, PI3K, p-AKT, AKT, p-GSK-3β, and GSK-3β. The next day, the blots were exposed to secondary antibodies for 1 h at room temperature and then captured with the Clinx Image Analysis System (CLiNX, China) using the enhanced chemiluminescence reagent and analyzed using Image J software. The antibodies used in WB analysis were detailed in Table S3.

**Immunoprecipitation (IP)**

Total protein was isolated from H9C2 cells using the cell lysis buffer for Western and IP. The IP was performed with an Immunoprecipitation Kit. After magnetic beads and sample preparation, the anti-NRF2 antibody and protein lysate were slowly rotated at 4 °C overnight. The second day, protein A/G agarose beads were added to each tube and rotated at 4 °C for 1 hour. The precipitates were collected and washed. Subsequently, the proteins were eluted with SDS sample buffer and then denaturized. The agarose beads were precipitated by centrifugation and then removed. The supernatant was analyzed by SDS-PAGE as previously described. The anti-ubiquitin antibody was applied to detect the ubiquitination level of NRF2. The antibodies used in IP analysis were detailed in Table S3.

**Mendelian randomization (MR) analysis**

A two-sample MR analysis was employed to explore the causal association between the substrate of ALOX5 and HF. Nine independent (clump with r^2^ < 0.01 and distance > 10,000 kb) single-nucleotide polymorphisms (SNPs) at the level of genome-wide significance (*P* < 5×10^−8^) were selected from genome-wide association study (GWAS) of the Cohorts for Heart and Aging Research in Genomic Epidemiology (CHARGE) Consortium in 8,631 individuals of European ancestry as instrumental variables for AA, as the previous studies indicated ^11^. Information on these SNPs was summarized in Table S5.

Heart failure summary statistics were provided by the Heart Failure Molecular Epidemiology for Therapeutic Targets (HERMES) Consortium, which conducted a large meta-analysis of GWAS involving 47,309 patients and 930,014 controls of European ancestry across 26 studies ^12^. HF cases included subjects with incident or prevalent HF, with the HF definition based on self-reported information, physician diagnosis, or the ICD-9 or ICD-10 codes for discharge diagnosis.

The fixed-effects inverse-variance-weighted (IVW) method served as the primary MR analysis to estimate the causal relationship between AA and HF. To ensure the robustness of our findings, complementary analyses, including the weighted median ^13^ and MR-Egger regression ^14^ methods, were performed. Several sensitivity analyses were thereafter performed. First, Cochran’s Q statistic was used to assess heterogeneity among the SNPs. A *P*-value for Cochran’s Q less than 0.05 was considered as the presence of heterogeneity. Second, the intercept from the MR-Egger regression was used to detect potential horizontal pleiotropy. An intercept *P*-value greater than 0.05 suggested no evidence of horizontal pleiotropy. Third, scatter plots were provided to visualize the relationship between the SNP effects on the exposure (AA) and the outcome (HF).

**Statistics**

Each experiment was independently repeated at least three times, with the sample size (n) detailed in the figure legends.

**References**

1 Davis, S. & Meltzer, P. S. GEOquery: a bridge between the Gene Expression Omnibus (GEO) and BioConductor. *Bioinformatics* **23**, 1846-1847 (2007).

2 Ritchie, M. E., Phipson, B., Wu, D., Hu, Y., Law, C. W., Shi, W. *et al.* limma powers differential expression analyses for RNA-sequencing and microarray studies. *Nucleic Acids Res* **43**, e47 (2015).

3 Yu, G., Wang, L. G., Han, Y. & He, Q. Y. clusterProfiler: an R package for comparing biological themes among gene clusters. *Omics* **16**, 284-287 (2012).

4 Jiang, Q., Chen, X., Tian, X., Zhang, J., Xue, S., Jiang, Y. *et al.* Tanshinone I inhibits doxorubicin-induced cardiotoxicity by regulating Nrf2 signaling pathway. *Phytomedicine* **106**, 154439 (2022).

5 Liu, Y., Zeng, L., Yang, Y., Chen, C., Wang, D. & Wang, H. Acyl-CoA thioesterase 1 prevents cardiomyocytes from Doxorubicin-induced ferroptosis via shaping the lipid composition. *Cell Death Dis* **11**, 756 (2020).

6 Mothe-Satney, I., Filloux, C., Amghar, H., Pons, C., Bourlier, V., Galitzky, J. *et al.* Adipocytes secrete leukotrienes: contribution to obesity-associated inflammation and insulin resistance in mice. *Diabetes* **61**, 2311-2319 (2012).

7 Kwak, H. J., Park, K. M., Choi, H. E., Lim, H. J., Park, J. H. & Park, H. Y. The cardioprotective effects of zileuton, a 5-lipoxygenase inhibitor, are mediated by COX-2 via activation of PKC delta. *Cell Signal* **22**, 80-87 (2010).

8 Alammari, A. H., Shoieb, S. M., Maayah, Z. H. & El-Kadi, A. O. S. Fluconazole Represses Cytochrome P450 1B1 and Its Associated Arachidonic Acid Metabolites in the Heart and Protects Against Angiotensin II-Induced Cardiac Hypertrophy. *J Pharm Sci* **109**, 2321-2335 (2020).

9 Xin, Y., Bai, Y., Jiang, X., Zhou, S., Wang, Y., Wintergerst, K. A. *et al.* Sulforaphane prevents angiotensin II-induced cardiomyopathy by activation of Nrf2 via stimulating the Akt/GSK-3ß/Fyn pathway. *Redox Biol* **15**, 405-417 (2018).

10 Lan, Y. J., Cheng, M. H., Ji, H. M., Bi, Y. Q., Han, Y. Y., Yang, C. Y. *et al.* Melatonin ameliorates bleomycin-induced pulmonary fibrosis via activating NRF2 and inhibiting galectin-3 expression. *Acta Pharmacol Sin* **44**, 1029-1037 (2023).

11 Zhang, T., Zhao, J. V. & Schooling, C. M. The associations of plasma phospholipid arachidonic acid with cardiovascular diseases: A Mendelian randomization study. *EBioMedicine* **63**, 103189 (2021).

12 Shah, S., Henry, A., Roselli, C., Lin, H., Sveinbjörnsson, G., Fatemifar, G. *et al.* Genome-wide association and Mendelian randomisation analysis provide insights into the pathogenesis of heart failure. *Nat Commun* **11**, 163 (2020).

13 Bowden, J., Davey Smith, G., Haycock, P. C. & Burgess, S. Consistent Estimation in Mendelian Randomization with Some Invalid Instruments Using a Weighted Median Estimator. *Genet Epidemiol* **40**, 304-314 (2016).

14 Bowden, J., Davey Smith, G. & Burgess, S. Mendelian randomization with invalid instruments: effect estimation and bias detection through Egger regression. *Int J Epidemiol* **44**, 512-525 (2015).

**Supplementary Tables**

**Supplementary Table S1. Patient characteristics.**

| Sample | Sex | Age (year) | Diagnosis | LVEF (%) |
| --- | --- | --- | --- | --- |
| HF1 | Male | 40 | Dilated cardiomyopathy | 27 |
| HF2 | Female | 69 | Dilated cardiomyopathy | 24 |
| HF3 | Male | 62 | Dilated cardiomyopathy | 30 |

HF, heart failure; LVEF, left ventricular ejection fraction.

**Supplementary Table S2. Information on the reagents used in this study.**

| **Reagents** | **Vendor or Source** | **Catalog** |
| --- | --- | --- |
| Dox | GlpBio | GC17567 |
| Zileuton | MCE | HY-14164 |
| Fer-1 | MCE | HY-100579 |
| 4% paraformaldehyde solution | Servicebio | G1101 |
| DMEM | Gibco | 11995073 |
| Fetal bovine serum | ExCell Bio | FSP500 |
| Arachidonic acid | TargetMol | T4129 |
| 5-HETE | GlpBio | GC40439 |
| LY294002 | MCE | HY-10108 |
| ML385 | MCE | HY-100523 |
| DAPI | Servicebio | G1012 |
| Cell lysis buffer for Western and IP | Beyotime | P0013 |
| Enhanced BCA protein assay kit | Beyotime | P0010 |
| Rat 5-hydroxyeicosatetraenoic acid ELISA Kit (5-HETE) | ABclonal | RK04790 |
| Mouse 5-hydroxyeicosatetraenoic acid ELISA Kit (5-HETE) | ABclonal | RK04791 |
| CCK-8 kit | GlpBio | GK10001 |
| Lipofectamine 3000 | Invitrogen | L3000015 |
| Opti-MEM | Gibco | 31985070 |
| LDH assay kit | Jiancheng | A020-2-2 |
| LDH Release Assay Kit | Beyotime | C0017 |
| GSH and GSSG Assay Kit | Beyotime | S0053 |
| Lipid Peroxidation MDA Assay Kit | Beyotime | S0131S |
| HBSS | Servicebio | G4204 |
| BODIPY™ 581/591 C11 | Invitrogen | D3861 |
| DHE probe | Beyotime | S0063 |
| Trizol | Servicebio | G3013 |
| cDNA Synthesis Kit | Servicebio | G3330 |
| Low ROX SYBR Green qPCR Master Mix | Servicebio | G3321 |
| RIPA Lysis Buffer | Beyotime | P0013B |
| Protease Inhibitor Cocktail | MCE | HY-K0010 |
| Phosphatase Inhibitor Cocktail I | MCE | HY-K0021 |
| PVDF membranes | Merck Millipore | ISEQ00010 |
| Enhanced chemiluminescence reagent | Biosharp | BL523B |
| Immunoprecipitation Kit | Beyotime | P2179 |

**Supplementary Table S3. Information on antibodies used in this study.**

| **Target antigen** | **Vendor or Source** | **Catalog** | **Dilution** | **Application** |
| --- | --- | --- | --- | --- |
| ALOX5 | Proteintech | 10021-1-Ig | 1:1000  1:100  1:100 | WB  IF  IHC |
| GAPDH | ABclonal | A19056 | 1:50000 | WB |
| Cardiac Troponin-T | Abcam | ab8295 | 1:200 | IF |
| Cy3-conjugated Goat anti-Rabbit IgG (H+L) | ABclonal | AS007 | 1:200 | IF |
| 488-conjugated Goat anti-Mouse IgG (H+L) | ABclonal | AS037 | 1:200 | IF |
| SLC7A11 | ABclonal | A2413 | 1:1000 | WB |
| GPX4 | ABclonal | A1933 | 1:1000 | WB |
| E2F1 | Proteintech | 12171-1-AP | 1:1000 | WB |
| EGR1 | ABclonal | A23424 | 1:1000 | WB |
| NRF2 | Proteintech | 16396-1-AP | 1:1000  1ug for 1mg total protein lysate | WB  IP |
| p-PI3K | CST | 4228 | 1:1000 | WB |
| PI3K | CST | 4292 | 1:1000 | WB |
| p-AKT | CST | 4060 | 1:1000 | WB |
| AKT | CST | 9272 | 1:1000 | WB |
| p-GSK-3β | Santa Cruz | Sc-373800 | 1:500 | WB |
| GSK-3β | Proteintech | 67329-1-Ig | 1:1000 | WB |
| anti-ubiquitin antibody | Santa Cruz | Sc-8017 | 1:200 | WB |

WB, Western Blot; IF, immunofluorescence; IHC, immunohistochemistry; IP, immunoprecipitation.

**Supplementary Table S4. Primer sequences used in RT-qPCR analysis.**

| **Gene name** | **Sequence (5'-3')** |
| --- | --- |
| *Gapdh* (human) | Forward: GGAGCGAGATCCCTCCAAAAT  Reverse: GGCTGTTGTCATACTTCTCATGG |
| *Nppb* (human) | Forward: TGGAAACGTCCGGGTTACAG  Reverse: CTGATCCGGTCCATCTTCCT |
| *Alox5* (human) | Forward: CTCAAGCAACACCGACGTAAA  Reverse: CCTTGTGGCATTTGGCATCG |
| *Gapdh* (mouse) | Forward: GGTTGTCTCCTGCGACTTCA  Reverse: GGTGGTCCAGGGTTTCTTACTC |
| *Alox5* (mouse) | Forward: GGGCTGTAGCGAGAAGCATC  Reverse: CACGGTGACATCGTAGGAGT |
| *Gapdh* (rat) | Forward: GGTGGACCTCATGGCCTACA  Reverse: CTCTCTTGCTCTCAGTATCCTTGCT |
| *Alox5* (rat) | Forward: GTCTGAGGTGTTCGGTATT  Reverse: TAGTGTTGATGGCAATGGT |
| *Nrf2* (rat) | Forward: ATTTGTAGATGACCATGAGTCGC  Reverse: TGTCCTGCTGTATGCTGCTT |
| *Egr1 (rat)* | Forward: GCTGGTGGAGACAAGTTAT |
| *E2f1* (rat) | Reverse: TGAGGATGAAGAGGTTGGA  Forward: GGAGAAGTCACGCTATGAG  Reverse: CTTGGCAATGAGTTGGATG |

RT-qPCR, real-time quantitative PCR.

**Supplementary Table S5. The genetic instruments for arachidonic acid and its genetic associations with heart failure.**

| SNP | Chr | Pos | Alleles (E/A) | EAF | F-statistic | SNP-exposure Associations | | | SNP-HF Associations | | |
| --- | --- | --- | --- | --- | --- | --- | --- | --- | --- | --- | --- |
|  |  |  |  |  |  | Beta^*^ | SE | P value | Beta | SE | P value |
| rs174547 | 11 | 61570783 | T/C | 0.680 | 4467 | 1.6909 | 0.0253 | 3.31E-971 | 0.0189 | 0.0083 | 0.0223 |
| rs472031 | 11 | 61638420 | A/G | 0.100 | 118 | 0.5097 | 0.0470 | 2.34E-27 | 0.0169 | 0.0141 | 0.2292 |
| rs760306 | 11 | 61724292 | T/C | 0.240 | 63 | -0.2808 | 0.0355 | 2.42E-15 | -0.0083 | 0.0090 | 0.3576 |
| rs12285167 | 11 | 61492039 | A/C | 0.199 | 51 | -0.2839 | 0.0399 | 1.05E-12 | -0.0043 | 0.0094 | 0.6508 |
| rs2903922 | 11 | 61929298 | A/T | 0.272 | 47 | -0.2327 | 0.0338 | 5.70E-12 | -0.0045 | 0.0087 | 0.6039 |
| rs1741 | 16 | 15130351 | C/G | 0.380 | 41 | -0.2008 | 0.0314 | 1.64E-10 | 0.0000 | 0.0086 | 0.9969 |
| rs259874 | 11 | 61811441 | A/G | 0.097 | 33 | -0.4120 | 0.0719 | 9.79E-09 | 0.0055 | 0.0127 | 0.6662 |
| rs3741259 | 11 | 61282350 | T/C | 0.104 | 30 | -0.3149 | 0.0576 | 4.50E-08 | 0.0111 | 0.0136 | 0.4166 |
| rs17663676 | 11 | 62201002 | T/C | 0.966 | 30 | -0.4688 | 0.0857 | 4.52E-08 | 0.0012 | 0.0200 | 0.9536 |

SNP, single nucleotide polymorphism; Chr, chromosome; Pos, position; E/A, effect/alternative alleles; EAF, effect allele frequency; F-statistics was calculated as follows: F =Beta^2^/SE^2^.

^*^ Change in percentage of total fatty acids per each additional copy of effect allele.

**Supplementary Figures**

**
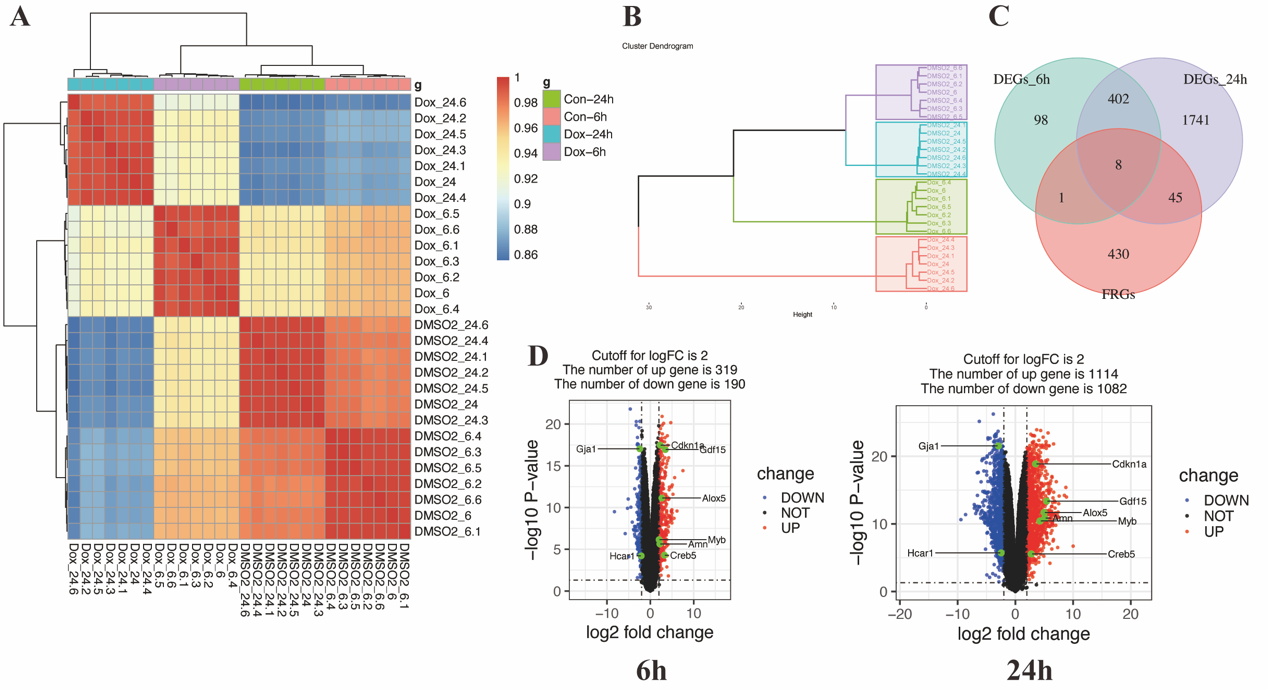
**

**Figure S1. RNA-sequencing analysis of the GSE166957 dataset.**

(A, B) Correlation analysis and cluster analysis were conducted to check whether the gene expression patterns were consistent within each group but distinct between groups. (C) Venn diagram identified 8 DEFRGs by intersecting the DEGs from NRCMs treated with Dox or DMSO for 6 hours or 24 hours with FRGs. (D) Volcano plots showing DEGs in NRCMs treated with Dox or DMSO for 6 hours or 24 hours. Genes that are upregulated are marked in red, while those that are downregulated are marked in blue (*P* < 0.05 and | log2 (fold change) | > 2). DEFRGs, differentially expressed ferroptosis-related genes; DEGs, differentially expressed genes; NRCMs, neonatal rat cardiomyocytes; Dox, doxorubicin; FRGs, ferroptosis-related genes.


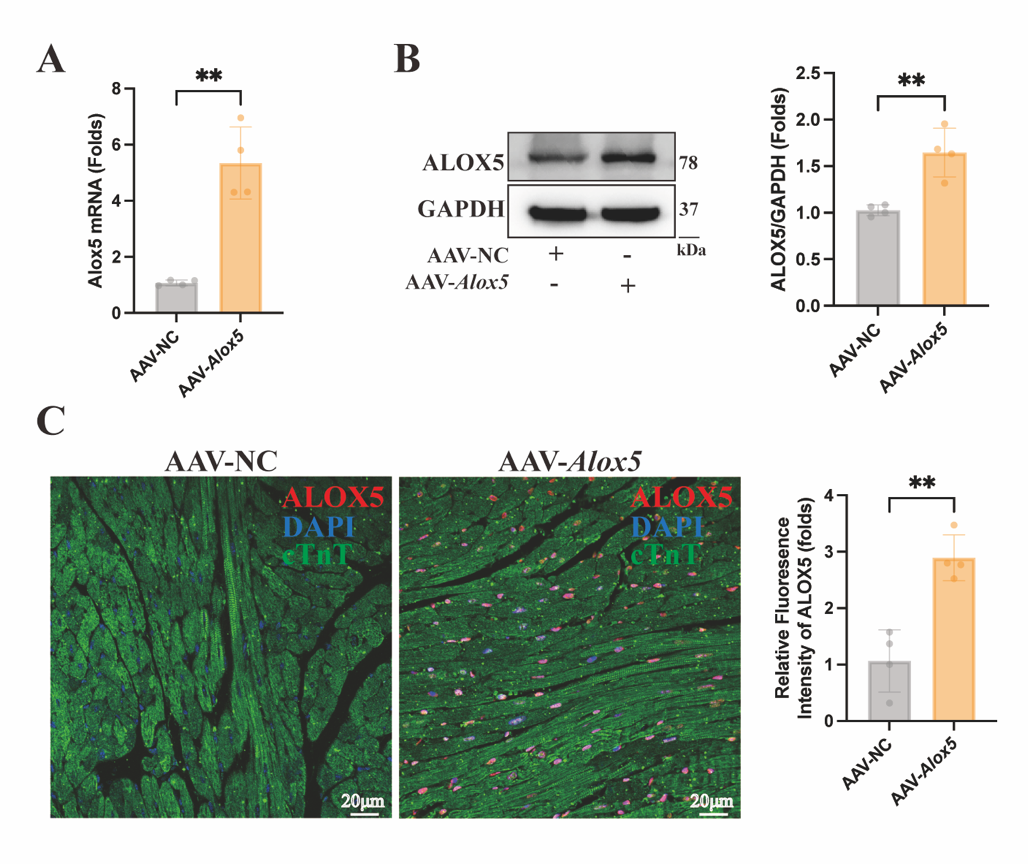


**Figure S2. The efficiency of cardiomyocyte-specific *Alox5* overexpression in vivo.**

(A) *Alox5* mRNA level in heart tissues (n = 4). (B) Representative WB images and quantitative analysis of ALOX5 protein expression in heart tissues (n = 4). (C) Representative immunofluorescence staining images and quantitative analysis of ALOX5 protein level in heart tissues (n = 4). **, *P* < 0.01. WB, Western Blot.


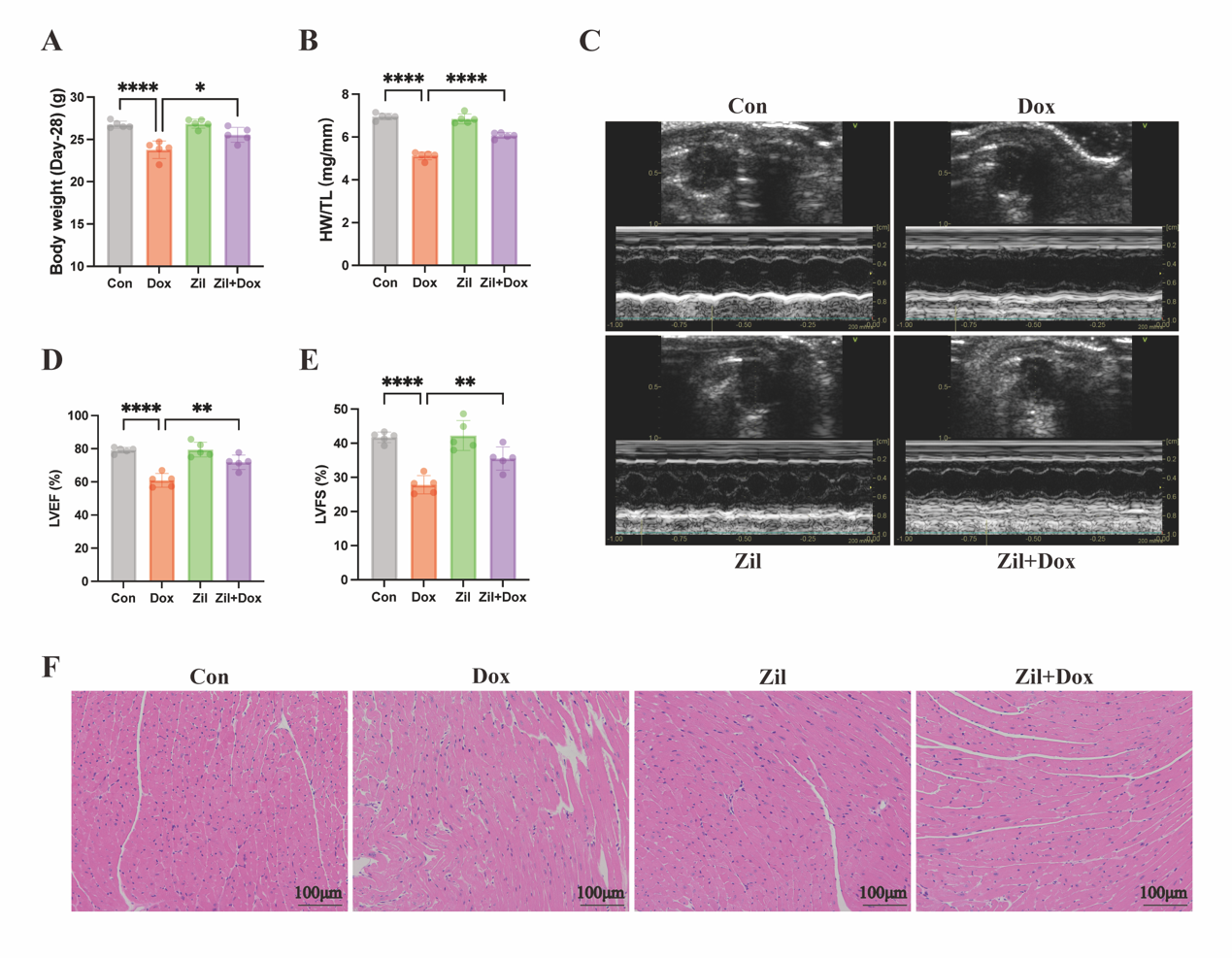


**Figure S3. ALOX5 inhibition ameliorates DIC in the in vivo DIC model established through tail vein injection of Dox.**

(A, B) Body weight and HW/TL of mice (n = 5). (C-E) Representative cardiac echocardiography images and quantitative analysis of LVEF and LVFS of mice (n = 5). (F) Representative HE staining images of heart tissues (n = 3). *, *P* < 0.05; **, *P* < 0.01; ****, *P* < 0.0001. DIC, doxorubicin-induced cardiomyopathy; Dox, doxorubicin; Zil, Zileuton; HW/TL, heart weight/tibia length; LVEF, left ventricular ejection fraction; LVFS, left ventricular fractional shortening; HE, Hematoxylin and Eosin.


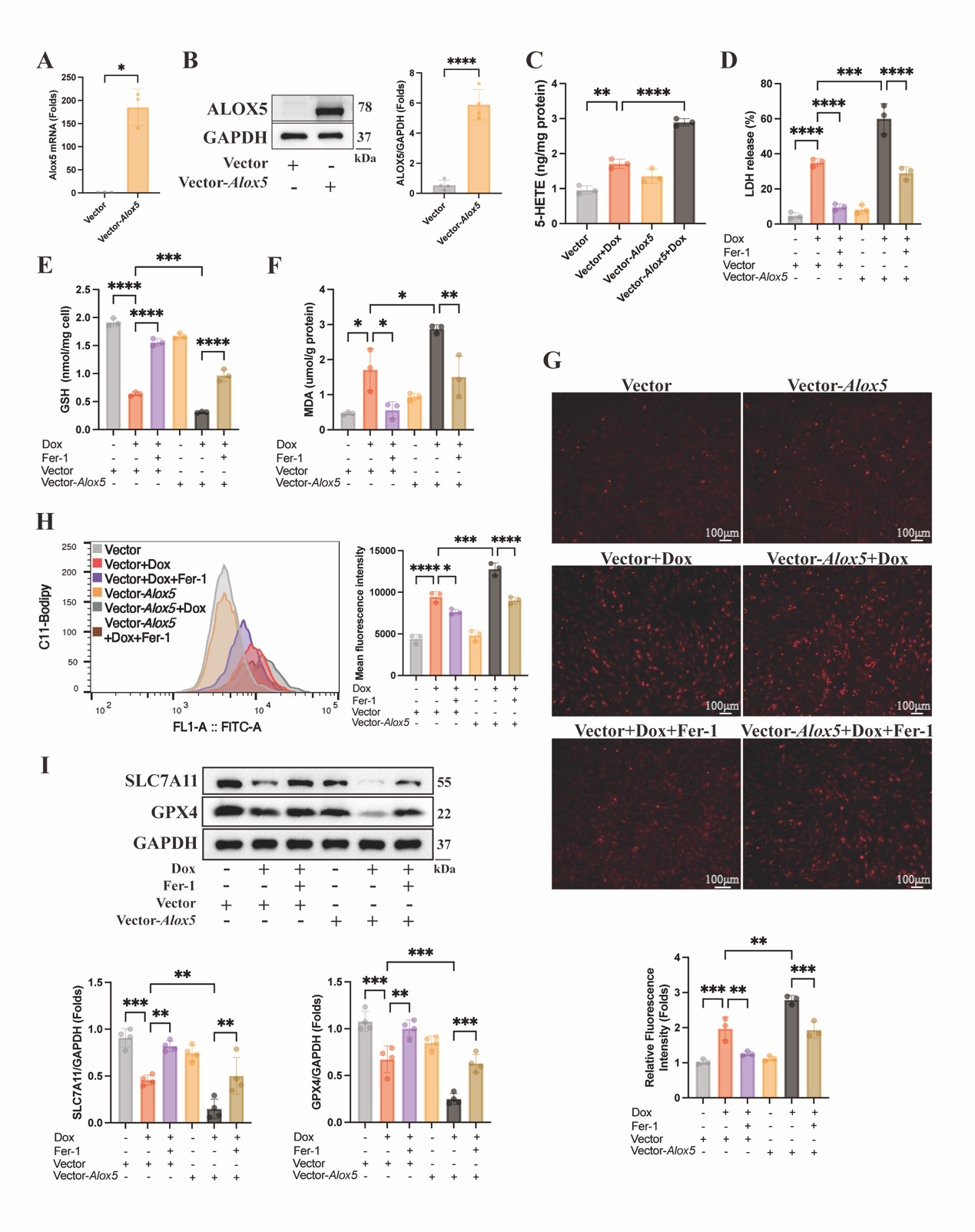
**Figure S4. *Alox5* overexpression aggravates DIC by promoting ferroptosis in vitro.**

(A) *Alox5* mRNA level in H9C2 cells (n = 3). (B) Representative WB images and quantitative analysis of ALOX5 protein expression in H9C2 cells (n = 4). (C) 5-HETE content in H9C2 cells (n = 3). (D-F) Quantitative analysis of the LDH release, GSH content, and MDA levels in H9C2 cells (n = 3). (G) Representative images and quantitative results of DHE staining of H9C2 cells (n = 3). (H) Flow cytometer analysis and quantitative results of C11-Bodipy staining (n = 3). (I) Representative WB images and quantitative analysis of protein expressions of SLC7A11 and GPX4 in H9C2 cells (n = 4). *, *P* < 0.05; **, *P* < 0.01; ***, *P* < 0.001; ****, *P* < 0.0001. DIC, doxorubicin-induced cardiomyopathy; 5-HETE, 5-hydroxyicosatetraenoic acid; Dox, doxorubicin; Fer-1, Ferrostatin-1; LDH, lactic dehydrogenase; GSH, glutathione; MDA, malondialdehyde; DHE, Dihydroethidium; WB, Western Blot.


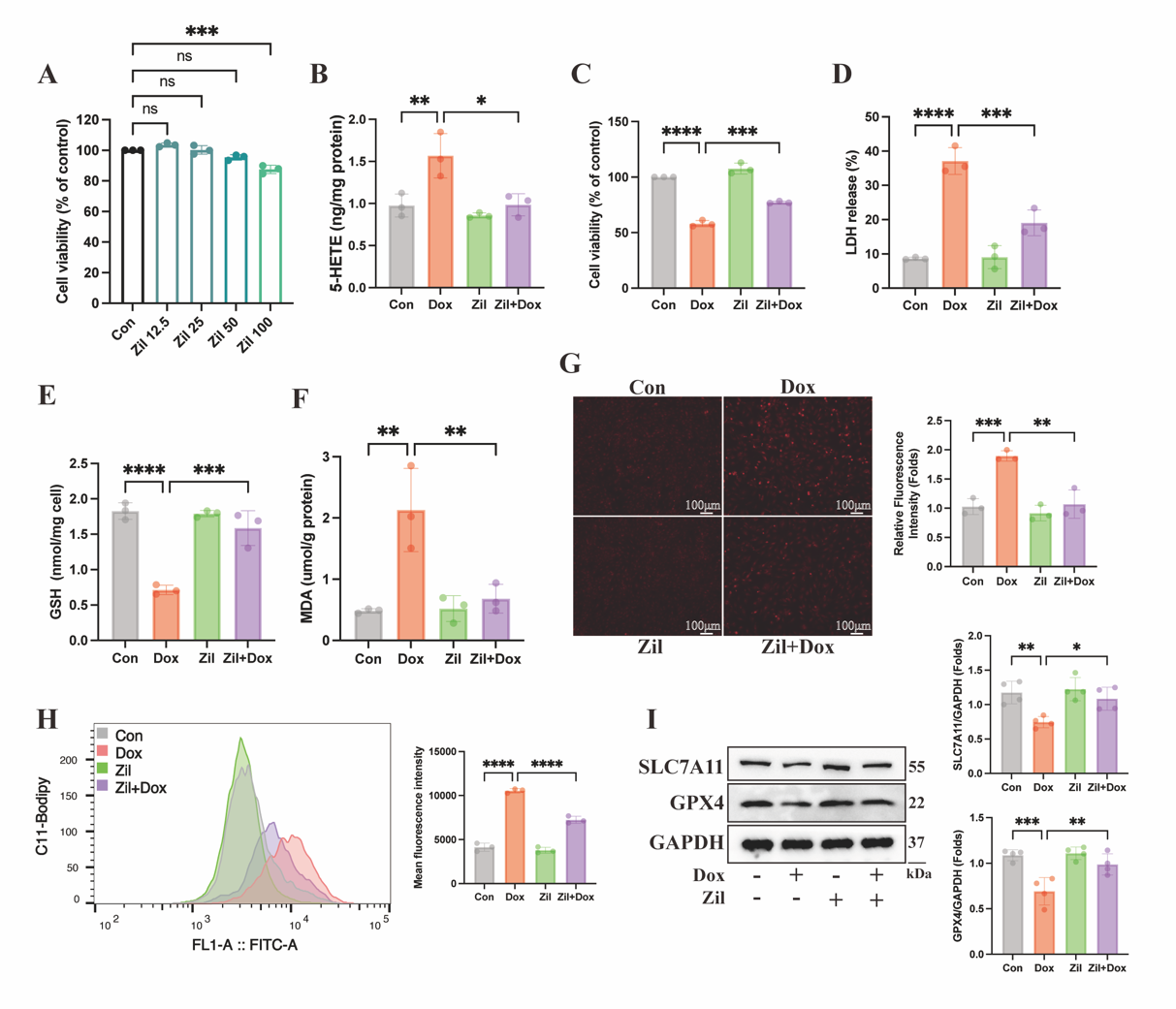


**Figure S5. ALOX5 inhibition attenuates DIC by suppressing ferroptosis in vitro.**

(A) CCK8 analysis of the viability of H9C2 cells after administrating Zil for 24 hours at different concentrations (n = 3). (B) 5-HETE content in H9C2 cells (n = 3). (C) CCK8 analysis of the effect of Zil on the viability of Dox-treated H9C2 cells (n = 3). (D-F) Quantitative analysis of the LDH release, GSH content, and MDA levels in H9C2 cells (n = 3). (G) Representative images and quantitative results of DHE staining of H9C2 cells (n = 3). (H) Flow cytometer analysis and quantitative results of C11-Bodipy staining (n = 3). (I) Representative WB images and quantitative analysis of protein expressions of SLC7A11 and GPX4 in H9C2 cells (n = 4). *, *P* < 0.05; **, *P* < 0.01; ***, *P* < 0.001; ****, *P* < 0.0001. DIC, doxorubicin-induced cardiomyopathy; Zil, Zileuton; Dox, doxorubicin; 5-HETE, 5-hydroxyicosatetraenoic acid; LDH, lactic dehydrogenase; GSH, glutathione; MDA, malondialdehyde; DHE, Dihydroethidium; WB, Western Blot.


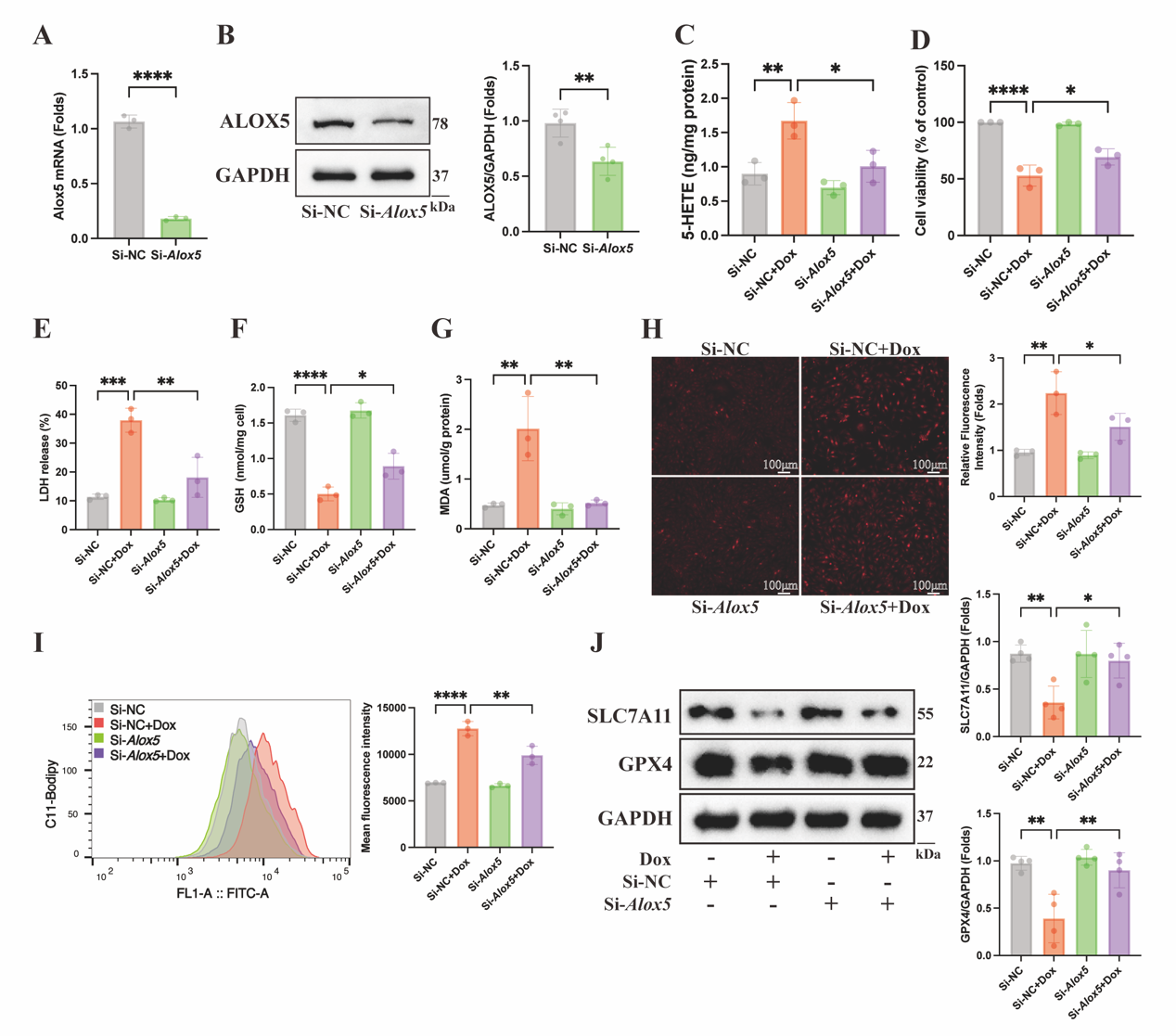


**Figure S6. *Alox5* knockdown attenuates DIC by inhibiting ferroptosis in vitro.**

(A) *Alox5* mRNA level in H9C2 cells (n = 3). (B) Representative WB images and quantitative analysis of ALOX5 protein expression in H9C2 cells (n = 4). (C) 5-HETE content in H9C2 cells (n = 3). (D) CCK8 analysis of the effect of *Alox5* knockdown on cell viability (n = 3). (E-G) Quantitative analysis of the LDH release, GSH content, and MDA levels in H9C2 cells (n = 3). (H) Representative images and statistical results of DHE staining of H9C2 cells (n = 3). (I) Flow cytometer analysis and quantitative results of C11-Bodipy staining (n = 3). (J) Representative WB images and quantitative analysis of protein expressions of SLC7A11 and GPX4 in H9C2 cells (n = 4). *, *P* < 0.05; **, *P* < 0.01; ***, *P* < 0.001; ****, *P* < 0.0001. DIC, doxorubicin-induced cardiomyopathy; Dox, doxorubicin; 5-HETE, 5-hydroxyicosatetraenoic acid; LDH, lactic dehydrogenase; GSH, glutathione; MDA, malondialdehyde; DHE, Dihydroethidium; WB, Western Blot.

**
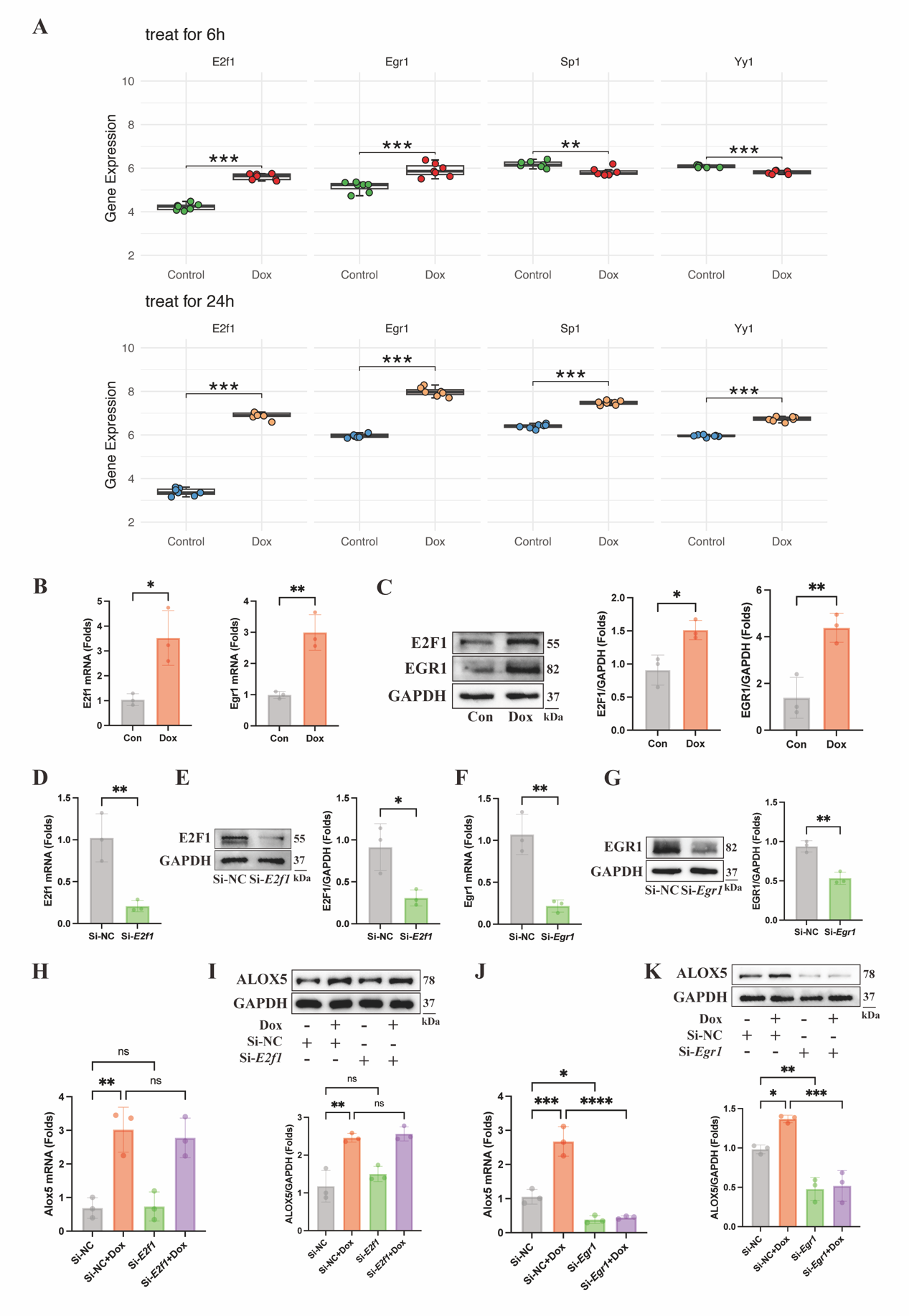
**

**Figure S7. Upregulation of ALOX5 in DIC is transcriptionally regulated by EGR1.**

(A) The expression levels of *E2f1*, *Egr1*, *Sp1*, and *Yy1* in NRCMs treated with Dox or DMSO for 6 hours or 24 hours. (B) *E2f1* and *Egr1* mRNA levels in H9C2 cells (n = 3). (C) Representative WB images and quantitative analysis of E2F1 and EGR1 protein expression in H9C2 cells (n = 3). (D, E) RT-qPCR and WB analysis of E2F1 expression in H9C2 cells (n = 3). (F, G) RT-qPCR and WB analysis of EGR1 expression in H9C2 cells (n = 3). (H-K) RT-qPCR and WB analysis of ALOX5 expression in H9C2 cells (n = 3). ns, no significance; *, *P* < 0.05; **, *P* < 0.01; ***, *P* < 0.001; ****, *P* < 0.0001. DIC, doxorubicin-induced cardiomyopathy; NRCMs, neonatal rat cardiomyocytes; Dox, doxorubicin; RT-qPCR, real-time quantitative PCR; WB, Western Blot.


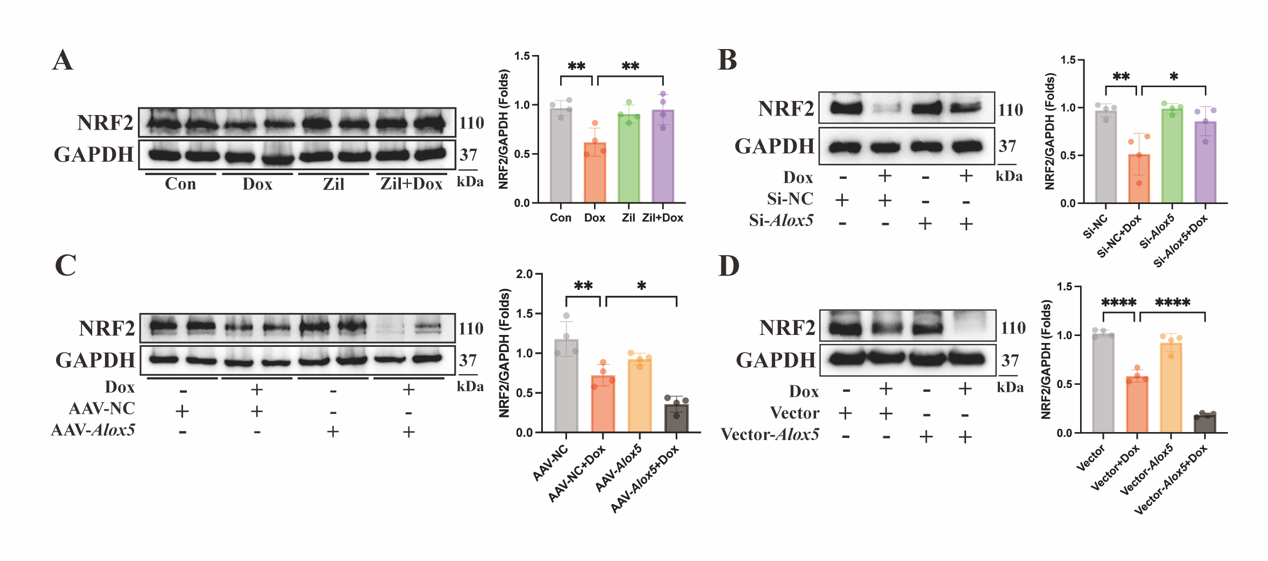


**Figure S8. The effect of modulating ALOX5 on the protein levels of NRF2 in vivo and in vitro.**

(A) Effect of ALOX5 inhibition with Zil on the protein level of NRF2 in heart tissues of mice (n = 4). (B) Effect of *Alox5* knockdown with siRNA on the protein level of NRF2 in H9C2 cells (n = 4). (C, D) Effect of *Alox5* overexpression on the protein level of NRF2 in heart tissues of mice and H9C2 cells (n = 4). *, *P* < 0.05; **, *P* < 0.01; ****, *P* < 0.0001. Dox, doxorubicin; Zil, Zileuton.

**
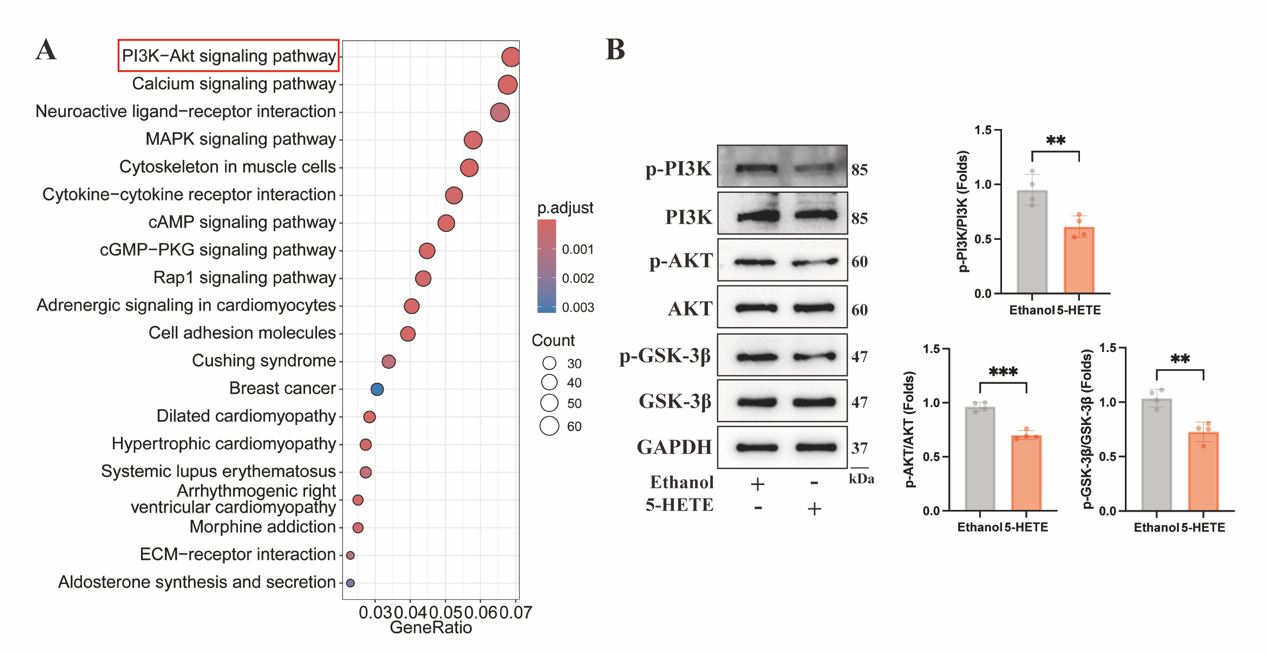
**

**Figure S9. The PI3K/AKT signaling pathway was enriched in DIC and 5-HETE inhibited the PI3K/AKT/GSK-3β pathway.**

(A) Enriched items in KEGG analysis using DEGs from NRCMs treated with Dox or DMSO for 24 hours. (B) Representative WB images and quantitative analysis of protein levels of p-PI3K, PI3K, p-AKT, AKT, p-GSK-3β, and GSK-3β in Ethanol or 5-HETE-treated H9C2 cells (n = 4). **, *P* < 0.01; ***, *P* < 0.001. DIC, doxorubicin-induced cardiomyopathy; KEGG, Kyoto Encyclopedia of Genes and Genomes; DEGs, differentially expressed genes; NRCMs, neonatal rat cardiomyocytes; Dox, doxorubicin; 5-HETE, 5-hydroxyicosatetraenoic acid; WB, Western Blot.


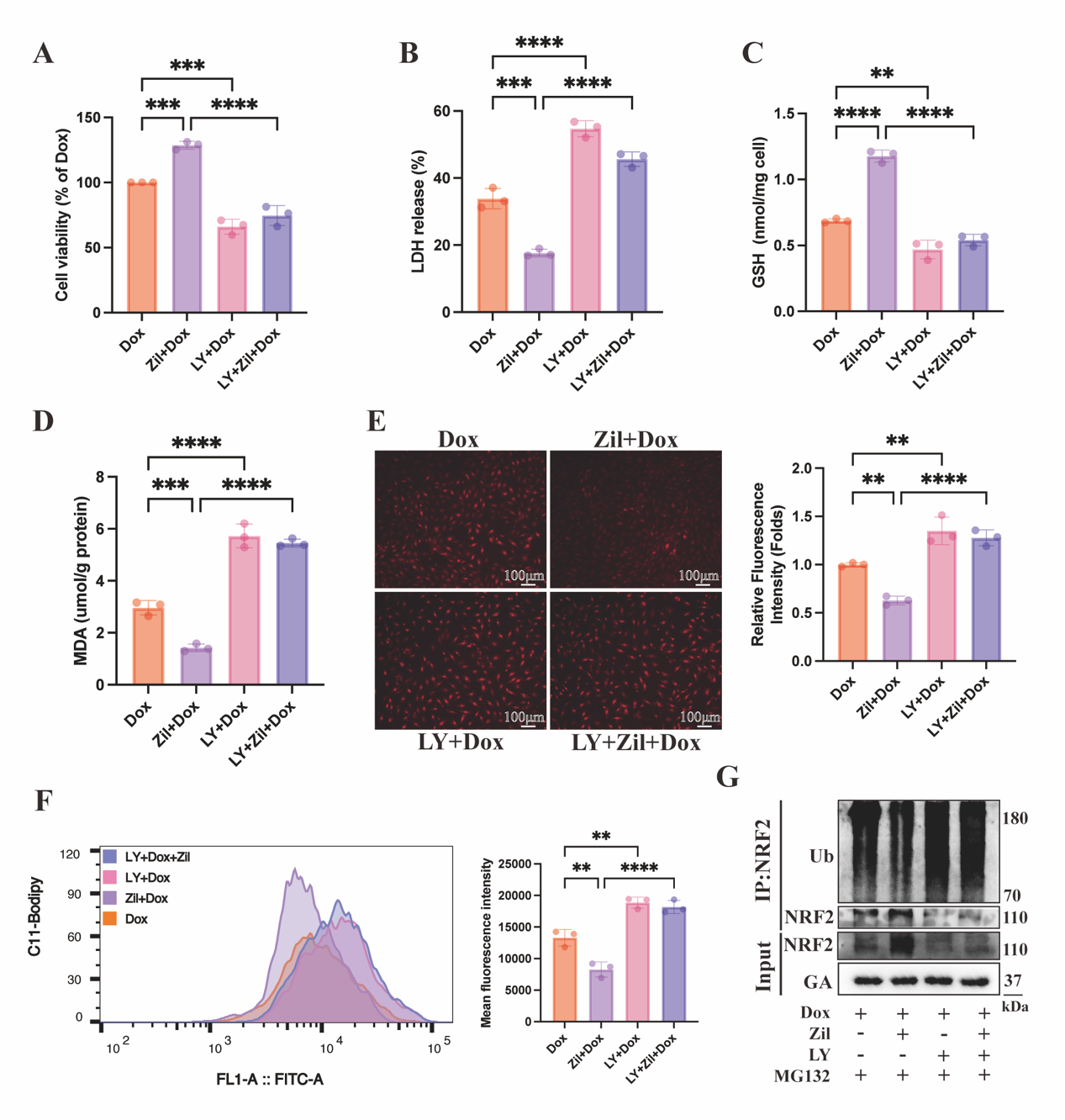


**Figure S10. Inhibition of PI3K/AKT activity abolished the protective effects of ALOX5 inhibition against DIC.**

(A) CCK8 analysis of the effect of LY294002 on cell viability (n = 3). (B-D) Quantitative analysis of the LDH release, GSH content, and MDA levels in H9C2 cells (n = 3). (E) Representative images and statistical results of DHE staining of H9C2 cells (n = 3). (F) Flow cytometer analysis and quantitative results of C11-Bodipy staining (n = 3). (G) Immunoprecipitation assay of cell lysates from Dox-treated H9C2 cells exposed to MG132 with or without Zil and LY294002 treatment, precipitated with anti-NRF2 antibody and then immunoblotted with anti-ubiquitin antibody (n = 3). **, *P* < 0.01; ***, *P* < 0.001; ****, *P* < 0.0001. DIC, doxorubicin-induced cardiomyopathy; Dox, doxorubicin; Zil, Zileuton; LY, LY294002; LDH, lactic dehydrogenase; GSH, glutathione; MDA, malondialdehyde; DHE, Dihydroethidium.

**
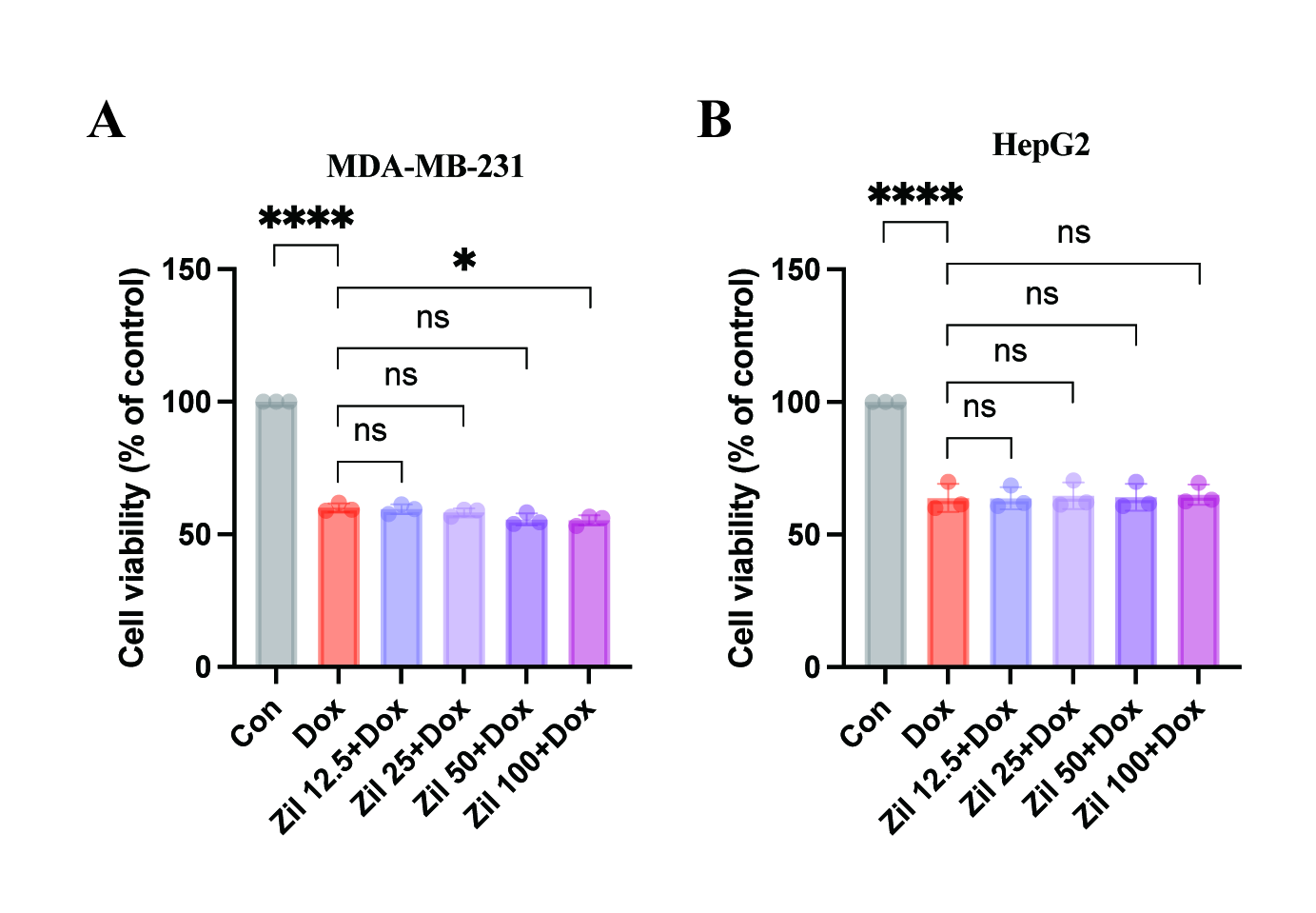
**

**Figure S11. ALOX5 inhibition did not affect the antitumor efficiency of Dox.**

(A) CCK8 analysis of the effect of Zil on the viability of Dox-treated MDA-MB-231 cells (n = 3). (B) CCK8 analysis of the effect of Zil on the viability of Dox-treated HepG2 cells (n = 3). ns, no significance; *, *P* < 0.05; ****, *P* < 0.0001. Dox, doxorubicin; Zil, Zileuton.
