# Supplementary material for: Arachidonate lipoxygenase 5 metabolism axis promoting ferroptosis: a potential druggable target for doxorubicin-induced cardiomyopathy
Source: Br J Cancer. 2026 Apr 6;134(11):1529–40. doi: 10.1038/s41416-026-03376-3 (PMC13183924; doi:10.1038/s41416-026-03376-3)

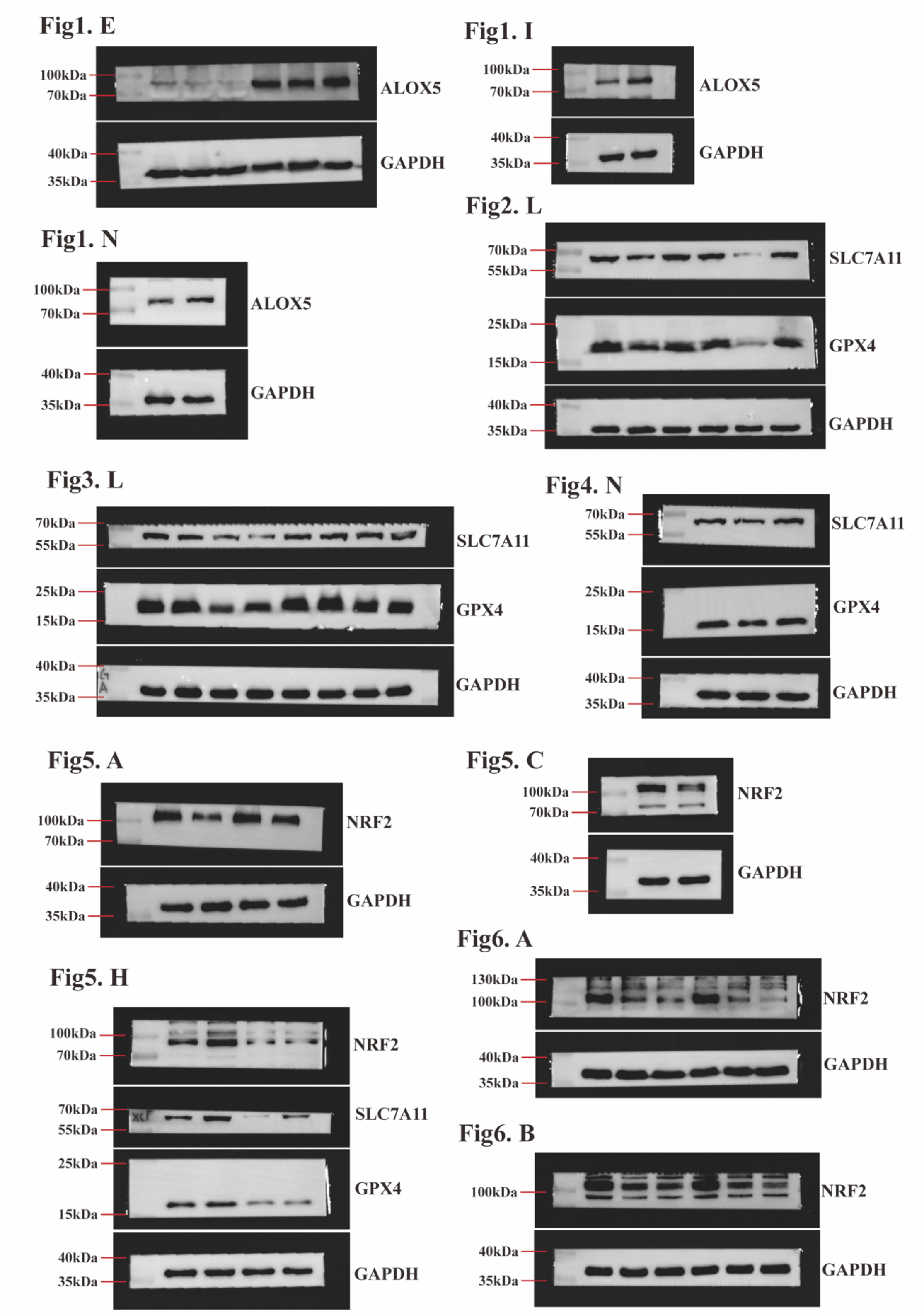
Fig1. E
100kDa
70kDa
ALOX5
40kDa
35kDa
GAPDH
Fig1. I
100kDa
70kDa
ALOX5
40kDa
35kDa
GAPDH
Fig1. N
100kDa
70kDa
ALOX5
40kDa
35kDa
GAPDH
Fig2. L
70kDa
55kDa
SLC7A11
25kDa
15kDa
GPX4
40kDa
35kDa
GAPDH
Fig3. L
70kDa
55kDa
SLC7A11
25kDa
15kDa
GPX4
40kDa
35kDa
GAPDH
Fig4. N
70kDa
55kDa
SLC7A11
25kDa
15kDa
GPX4
40kDa
35kDa
GAPDH
Fig5. A
100kDa
70kDa
NRF2
40kDa
35kDa
GAPDH
Fig5. C
100kDa
70kDa
NRF2
40kDa
35kDa
GAPDH
Fig5. H
100kDa
70kDa
NRF2
70kDa
55kDa
SLC7A11
25kDa
15kDa
GPX4
40kDa
35kDa
GAPDH
Fig6. A
130kDa
100kDa
NRF2
40kDa
35kDa
GAPDH
Fig6. B
100kDa
NRF2
40kDa
35kDa
GAPDH

Fig6. C

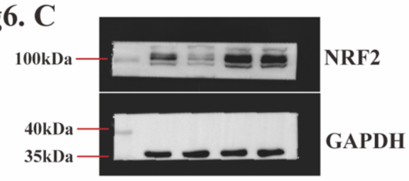

Fig6. D

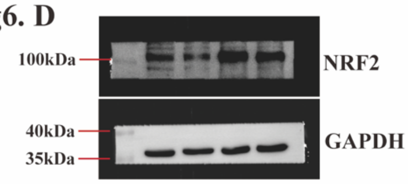

Fig6. F

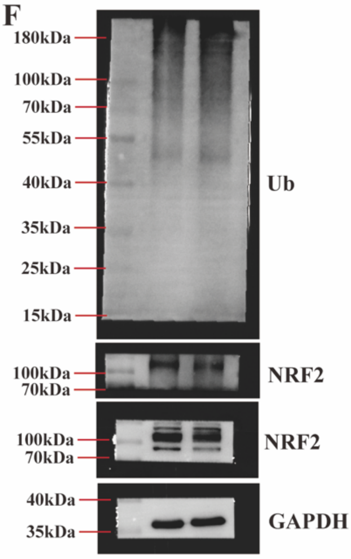

Fig6. H

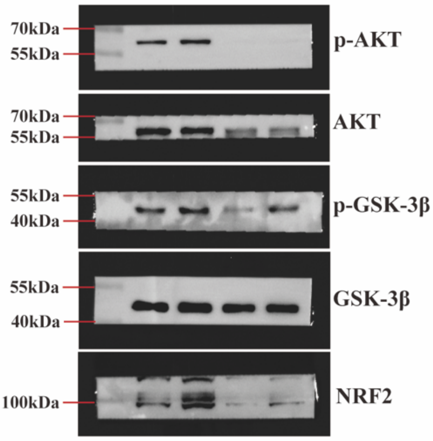

Fig6. E

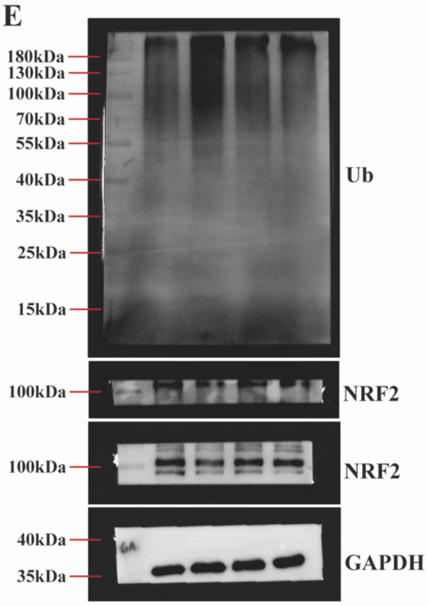

Fig6. G

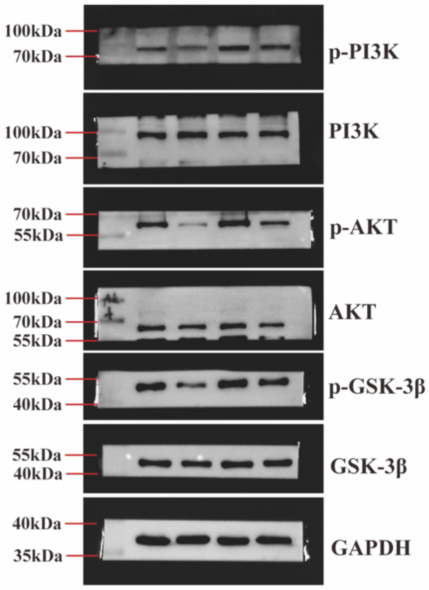

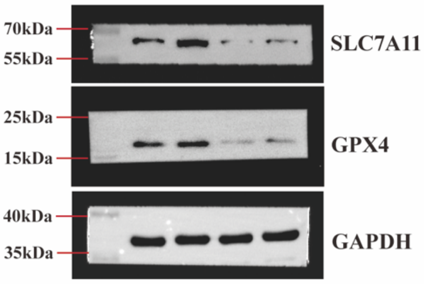

FigS2. B

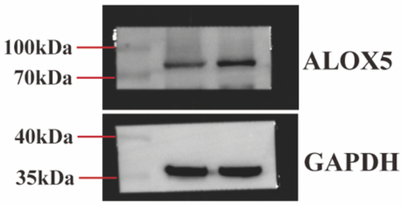

FigS4. B

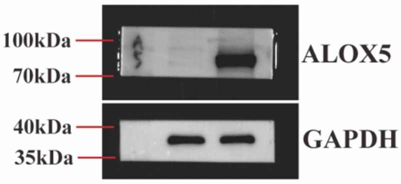

FigS4. I

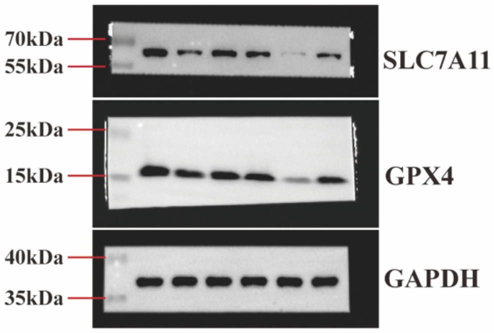

FigS5. I

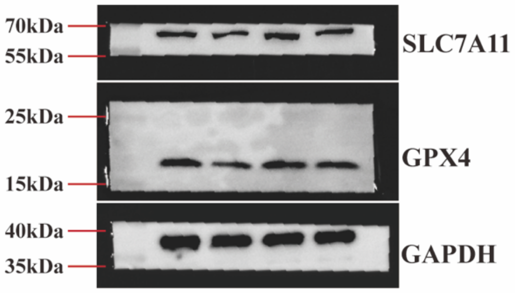

FigS6. B

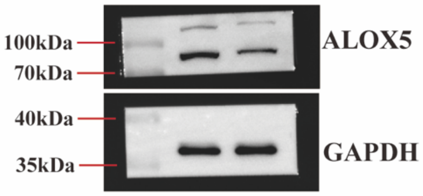

FigS6. J

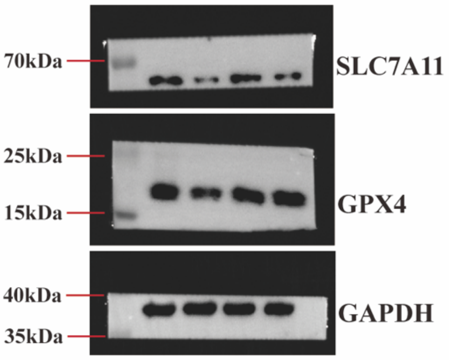

FigS7. C

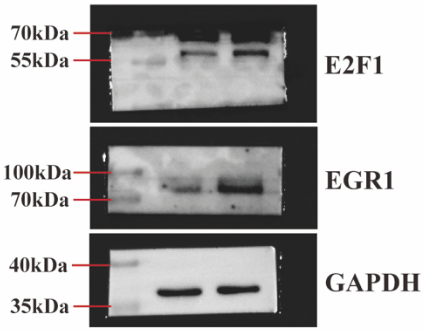

FigS7. E

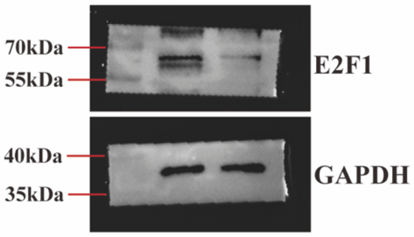

FigS7. G

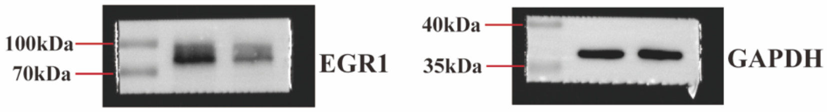

FigS7. I

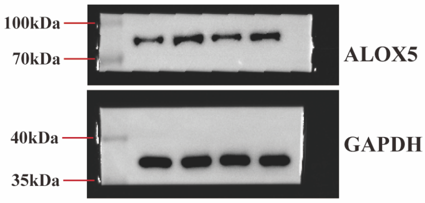

FigS7. K

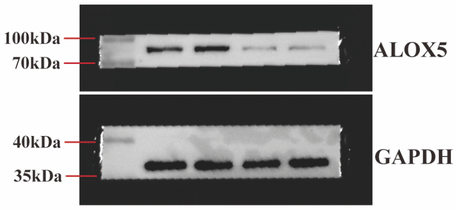

FigS8. A

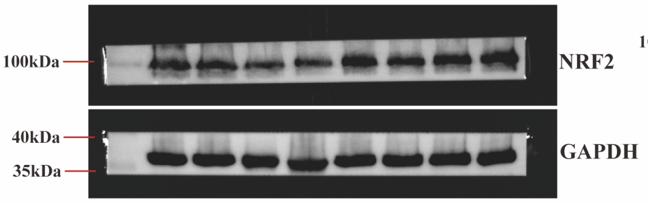

FigS8. B

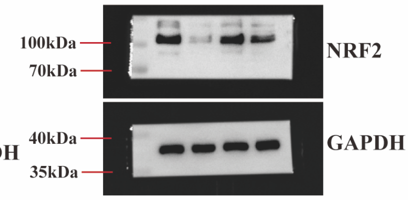

FigS8. C

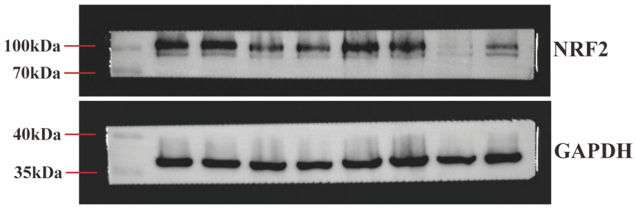

FigS8. D

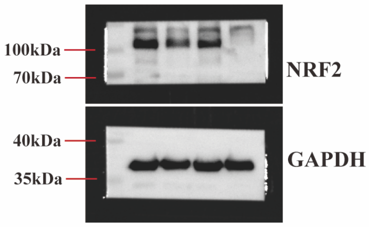

FigS9. B

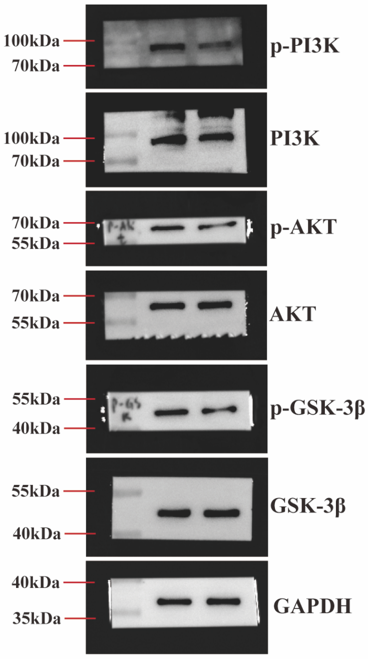

FigS10. G

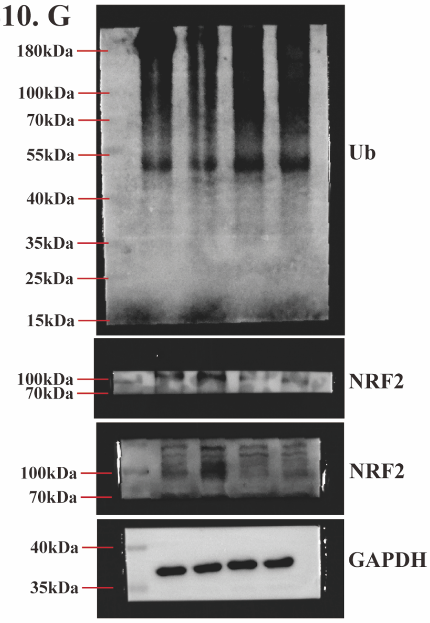

Supplement: Supplementary file 2 — Supplementary materials-Original Western Blot images [file 41416_2026_3376_MOESM2_ESM.pdf]
